# Chromosome-level genome assembly for the Aldabra giant tortoise enables insights into the genetic health of a threatened population

**DOI:** 10.1093/gigascience/giac090

**Published:** 2022-10-12

**Authors:** F Gözde Çilingir, Luke A'Bear, Dennis Hansen, Leyla R Davis, Nancy Bunbury, Arpat Ozgul, Daniel Croll, Christine Grossen

**Affiliations:** Department of Evolutionary Biology and Environmental Studies, University of Zurich, Zurich 8057, Switzerland; Seychelles Islands Foundation, Victoria, Republic of Seychelles; Zoological Museum, University of Zurich, Zurich 8006, Switzerland; Indian Ocean Tortoise Alliance, Ile Cerf, Victoria, Republic of Seychelles; Zoo Zürich, Zurich 8044, Switzerland; Seychelles Islands Foundation, Victoria, Republic of Seychelles; Centre for Ecology and Conservation, College of Life and Environmental Sciences, University of Exeter, Penryn, Cornwall, TR10 9FE, UK; Department of Evolutionary Biology and Environmental Studies, University of Zurich, Zurich 8057, Switzerland; Institute of Biology, University of Neuchâtel, Neuchâtel 2000, Switzerland; Department of Evolutionary Biology and Environmental Studies, University of Zurich, Zurich 8057, Switzerland

**Keywords:** Aldabrachelys gigantea, conservation management, rewilding, genome assembly, HiFi sequencing, Hi-C sequencing, reference genome

## Abstract

**Background:**

The Aldabra giant tortoise (*Aldabrachelys gigantea*) is one of only two giant tortoise species left in the world. The species is endemic to Aldabra Atoll in Seychelles and is listed as Vulnerable on the International Union for Conservation of Nature Red List (v2.3) due to its limited distribution and threats posed by climate change. Genomic resources for *A. gigantea* are lacking, hampering conservation efforts for both wild and *ex situ*populations. A high-quality genome would also open avenues to investigate the genetic basis of the species’ exceptionally long life span.

**Findings:**

We produced the first chromosome-level *de novo* genome assembly of *A. gigantea* using PacBio High-Fidelity sequencing and high-throughput chromosome conformation capture. We produced a 2.37-Gbp assembly with a scaffold N50 of 148.6 Mbp and a resolution into 26 chromosomes. RNA sequencing–assisted gene model prediction identified 23,953 protein-coding genes and 1.1 Gbp of repetitive sequences. Synteny analyses among turtle genomes revealed high levels of chromosomal collinearity even among distantly related taxa. To assess the utility of the high-quality assembly for species conservation, we performed a low-coverage resequencing of 30 individuals from wild populations and two zoo individuals. Our genome-wide population structure analyses detected genetic population structure in the wild and identified the most likely origin of the zoo-housed individuals. We further identified putatively deleterious mutations to be monitored.

**Conclusions:**

We establish a high-quality chromosome-level reference genome for *A. gigantea* and one of the most complete turtle genomes available. We show that low-coverage whole-genome resequencing, for which alignment to the reference genome is a necessity, is a powerful tool to assess the population structure of the wild population and reveal the geographic origins of *ex situ* individuals relevant for genetic diversity management and rewilding efforts.

## Background

As human activities drive our planet into its sixth mass extinction [1], genomic technologies are an important tool for conservation researchers. The establishment of reference-quality genomes for threatened species makes key contributions to the study of common genetic health issues. These include elucidating the full spectrum of genomic diversity; accurately quantifying inbreeding, mutation load, and introgression; detecting hybridization; and identifying adaptive variation in the face of rapidly changing environments [2]. The number of available reference genomes for nonmodel species has been increasing due to ongoing efforts in several global genome consortia, such as the Earth Biogenome Project [3], the Vertebrate Genomes Project [4, 5], and the Global Invertebrate Genomics Alliance [6]. However, available reference genomes of nonmodel species are not homogeneously distributed across the tree of life. Only three reference genomes represent the Testudinidae family (tortoises, overall 44 species [7]) from two genera, with two genomes being annotated and only one assembled to chromosome level. Tortoises have been integral components of global ecosystems for about 220 million years [8], contributing to seed dispersal, nutrient and mineral cycling, and carbon storage [9]. Over their long evolutionary history, giant tortoises, in particular, have evolved a life history characterized by delayed maturity, extended reproductive lives, and extreme longevity [10].

**Figure 1:**
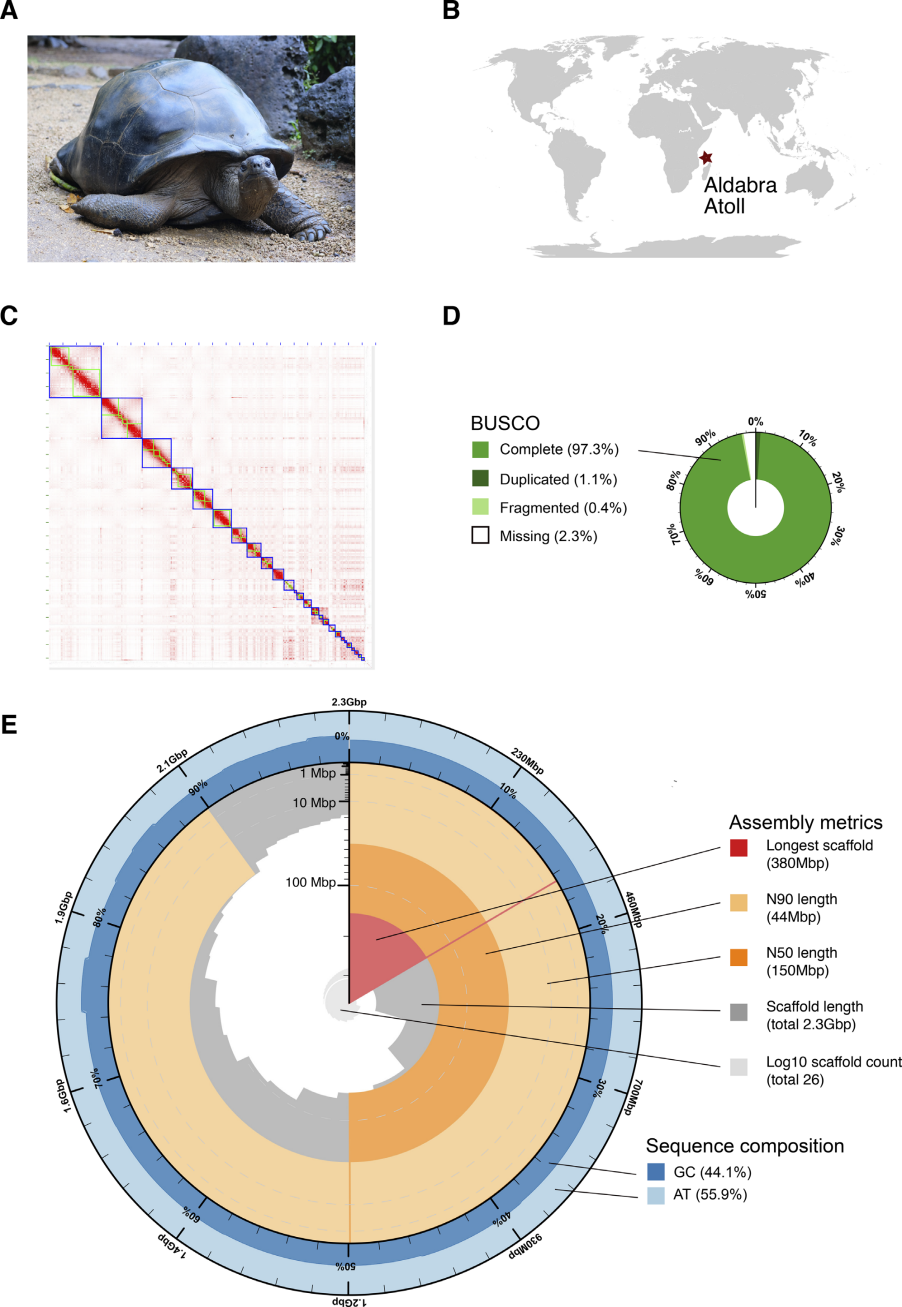
(A) A female *Aldabrachelys gigantea* resting at La Vanille Nature Park, Mauritius. (B) World map showing the location of Aldabra Atoll. (C) Hi-C contact map of the chromosome-level assembled *A. gigantea* reference genome. Blue boxes represent assembled pseudo-chromosomes and green boxes represent assembled scaffolds that constitute pseudo-chromosomes. (D) BUSCO completeness scores for the Sauropsida dataset and (E) assembly metrics (including length of the longest scaffold N50 and N90) and sequence composition (GC content) of the chromosome-level *A. gigantea* genome.

Currently, there are only two extant giant tortoise taxa, both of which face extinction threats [7]. Galápagos giant tortoises (*Chelonoidis niger* and subspecies thereof, formerly *Chelonoidis niger* species complex) are native to the Galápagos Islands in the Eastern Pacific Ocean, and taxa of this group are listed as vulnerable, endangered, or extinct according to the International Union for Conservation of Nature (IUCN) Red List (v2.3) [11]. Aldabra giant tortoises (*Aldabrachelys gigantea*) (Fig. 1A) are endemic to Aldabra Atoll in the Western Indian Ocean (Fig. 1B). Due to their extremely limited distribution in the wild and the threats posed by climate change, the species is listed as vulnerable on the IUCN Red List v2.3 [12]. Genomes of giant tortoises may harbor clues to their exceptional life history traits such as long life span [13] and gigantism [14–16]. Assessing genome-wide variation within species, including deleterious mutation load, will critically improve conservation management programs [17]. The recently established reference genome for one of the Galápagos giant tortoises, *Chelonoidis niger abingdonii*, revealed insights into potentially aging, disease-causing, and cancer-related gene functions by analyzing gene content evolution among tortoises [18]. For Aldabra giant tortoises, however, only short-read sequencing data are available from the same study [18].

*A. gigantea* (NCBI:txid167804) have been successfully used in rewilding projects on several Western Indian Ocean Islands, whose endemic giant tortoise species are now extinct [19]. The introduced populations act as ecological replacements for the extinct species and take a central role in shaping and sustaining large-scale vegetation dynamics as the largest frugi- and herbivore [20–23]. *A. gigantea* has been introduced to three islands belonging to Mauritius, including Ile aux Aigrettes, Round Island, and Rodrigues [24]. Monitoring the effectiveness of these rewilding projects will be crucial for catalyzing larger projects in Madagascar [25]. *A. gigantea* rewilding programs require genomic information and monitoring to minimize founder effects and maximize genetic variation in newly introduced populations [26]. Finally, uncertainties exist about the existence of additional *Aldabrachelys* lineages, as well as the number and taxonomic status of extinct lineages [7] due to weak morphological resolution and low-resolution genetic marker sets [27, 28].

Here, we present the first high-quality chromosome-level genome of *A. gigantea* using PacBio high-fidelity (HiFi) sequencing and chromosome conformation capture (Hi-C) sequencing for scaffolding. We assessed the utility of the reference genome by performing low-coverage whole-genome resequencing for 32 tortoises (30 wild and two zoo-housed individuals). We inferred the genetic structure of the wild population and the likely origin of zoo-housed individuals.

## Data Description

### Genome sequencing and assembly

#### DNA extraction, PacBio library preparation, and sequencing

In December 2020, during routine veterinary blood sampling, a subsample of approximately 3 mL of whole blood was collected from a female *A. gigantea* (named Hermania) living in the Zurich Zoo since 1955. Because blood was subsampled during a routine veterinary blood sampling, no additional ethical approval was required. Whole blood was taken from the animal's dorsal tail vein and stored on ice in a heparin-coated blood collection tube. DNA extraction was carried out at the Genetic Diversity Center, ETH, Zurich, according to the manufacturer's instructions of the MagAttract® High Molecular Weight DNA (HMW) Kit (Qiagen, Hilden, Germany), with a single modification: instead of using 200 µL whole blood as suggested for blood samples with nonnucleated red blood cells, a total of 50 µL whole blood was used. The purified DNA was eluted in 200 µL molecular-grade water. Subsequent steps, including genomic DNA (gDNA) quality control, PacBio HiFi library preparation, and sequencing, were carried out at the Functional Genomic Center Zurich, ETH.

The input HMW genomic DNA concentration was measured using a Qubit Fluorometer (Thermo Fisher, Waltham, Massachusetts, United States), and the DNA integrity was checked on a Femto Pulse Device (Agilent, Santa Clara, California, United States). The HiFi library preparation started with 14 μg HMW DNA. The PacBio HiFi library was produced using the SMRTbell® Express Template Prep Kit 2.0 (Pacific Biosciences, Menlo Park, California, United States), according to the manufacturer's instructions. Briefly, the DNA sample was mechanically sheared to an average size of 20 Kbp using a Megaruptor 3 Device (Diagenode, Liege, Belgium). A Femto Pulse gDNA analysis assay (Agilent) was used to assess the resulting fragment size distribution. The sheared DNA sample was DNA damage-repaired and end-repaired using polishing enzymes. PacBio sequencing adapters were ligated to the DNA template. A Blue Pippin device (Sage Science, Beverly, Massachusetts, United States) was used to size-select fragments >15 Kbp. The size-selected library was quality inspected and quantified using a Femto Pulse gDNA analysis assay (Agilent) and a Qubit Fluorometer (Thermo), respectively. The SMRT® bell-Polymerase Complex was prepared using the Sequel® II Binding Kit 2.0 and Internal Control 1.0 (Pacific Biosciences) and sequenced on a PacBio Sequel II instrument using the Sequel II Sequencing Kit 2.0 (Pacific Biosciences). In total, two Sequel II SMRT Cells 8 M (Pacific Biosciences) were run, taking one movie of 30 hours per cell. This yielded 49.4 Gbp of HiFi reads with a mean read length of 22.8 Kbp, which corresponds to approximately 20.8× coverage of the genome (NCBI SRA: SRR18672579) (Table 1).

**Table 1:** Summary of the genomic data produced in this study.

*Aldabrachelys gigantea* reference genome sequencing, assembly, and validation	
NCBI BioProject	PRJNA822095
**Draft genome sequencing**	
PacBio SMRT II HiFi data (Gb)	192
HiFi reads NCBI SRA Accession	SRR18672579
**Hi-C scaffolding**	
Illumina NovaSeq 6000 data (Gb)	196
Hi-C reads SRA Accession	SRR18673000
**Chromosome-level genome assembly (AldGig_1.0)**	
Assembled genome size (Gb)	2.37
Scaffold N50 (Mb)	148.6
No. of scaffolds	719
Contig N50 (Mb)	61.5
No. of contigs	422
BUSCO completeness (sauropsida_odb10)	97.3% complete, 0.4% fragmented, 2.3% missing
**Mitochondrial genome assembly**	
Assembled genome size (bp)	16,467
**Genome annotation**	
PacBio SMRT IsoSeq data (Gb)	1.1
IsoSeq reads NCBI SRA Accession	SRR18674283
No. of predicted protein-coding genes	23,953
No. of functionally annotated genes	22,554
Mean gene length (bp)	39,458
BUSCO completeness (sauropsida_odb10)	91.9% complete, 2.3% fragmented, 5.8% missing
DOI for annotations doi.org/10.5281/zenodo.6528994	
**Low coverage whole-genome resequencing**	
Illumina NovaSeq 6000 data (Gb)	202
NCBI SRA Accessions	SRR14611971-SRR18674101

#### Nuclear genome assembly, contamination scan, and evaluation

The consensus circular sequences per each Sequel II SMRT Cell (Pacific Biosciences) were filtered for adapter contamination with HiFiAdapterFilt v2.0.0 [29, 30] (-l 44, -m 97). Overall, 0.008% of the HiFi reads were filtered out. Genome size and heterozygosity rate were estimated based on the 17-mer frequency of the cleaned HiFi reads with GCE v1.0.2 (GCE, RRID:SCR_017332) [31, 32]. Our results indicate that *A. gigantea* has an estimated genome size of 2.37 Gbp (Supplementary Material S1) and low heterozygosity of 0.072% (corresponding to 0.72 single-nucleotide polymorphisms [SNPs] per 1 Kbp). This heterozygosity is consistent with recent estimates based on Illumina, San Diego, California, United States resequencing (0.78 SNPs per 1 Kbp [18]). The heterozygosity is also in the range of other endangered taxa, such as the Amur tiger (*Panthera tigris altaica*) (0.49 SNPs per 1 Kbp [33]), mountain gorilla (*Gorilla beringei beringei*) (0.65 SNPs per 1 Kbp [34]), or giant panda (*Ailuropoda melanoleuca*) (1.35 SNPs per 1 Kbp [35]), but higher than in some endangered turtle species such as the Pinta Island tortoise (*Chelonoidis niger abingdonii*) (0.13 SNPs per 1 Kbp [18]) and Reeves' Turtle (*Mauremys reevesii*) (0.60 SNPs per 1 Kbp [36]).

The reads were then assembled with the default parameters of HiCanu v2.1.1 (Canu, RRID:SCR_015880) [37, 38], Improved Phased Assembler v1.3.2 (IPA HiFi Genome Assembler, RRID:SCR_021966) [39], and Hifiasm v0.15.5 (Hifiasm, RRID:SCR_021069) [40, 41]. Additionally, an option for assembling inbred/homozygous genomes (-l 0) within Hifiasm [40, 41] was also tested. Main contiguity statistics were calculated with QUAST v5.0.2 (QUAST, RRID:SCR_001228) [42, 43]. The subsequent analyses were performed with the draft assembly obtained via Hifiasm [40, 41] with default parameters (-k 51, -a 4, -l 3, -s 0.75) because it provided the most contiguous and complete assembly with 483 contigs and an N50 of 61.5 Mbp (Supplementary Material S2).

Scanning for contaminant contigs in the draft assembly was performed by following three approaches. First, the draft assembly was split into 5-Kbp segments using SeqKit v0.16.1 (SeqKit, RRID:SCR_018926) [44, 45]. Each segment was searched against the full NCBI nonredundant protein database by running diamond v2.0.9 (DIAMOND, RRID:SCR_016071) [46, 47], a tool that performs protein alignments against reference databases, with the blastx (BLASTX, RRID:SCR_001653) option. We considered a segment to be a likely contaminant based on the blast bitscore (>30), e-value (>0.0001), and the segment's GC content (>70%). None of the blastx hits passed any of these cutoffs, and hence none of them was considered a significant match and potential contaminant. Second, we assessed *k*-mer profiles of the most probable sources of contamination: the human genome (NCBI RefSeq: GCF_000001405.39) and the *A. gigantea* mitochondrial genome (NCBI RefSeq: NC_028438.1). The average *k*-mer frequency of each contig in the draft assembly was compared with the potential contamination source using the tool *sect* in the software KAT v2.4.1 (KAT, RRID:SCR_016741) [48, 49]). Less than 0.01% of all contigs in the draft assembly showed *k*-mer statistics indicative of potential contamination (a validated *k*-mer coverage >1) by either source. Third, the previously published *A. gigantea* whole-genome resequencing dataset (NCBI SRA: SRX4741543) [18] was mapped against our assembly with BWA-MEM v0.7.17 [50]. The read coverage profile was examined with Qualimap v2.2.1 (QualiMap, RRID:SCR_001209) [51, 52]. The resequencing dataset had 27× coverage; therefore, we discarded contigs from the assembly with less than 10× or more than 100× aligned read depth. With all contaminant filtering steps combined, 62 contigs were removed from the assembly, resulting in a final set of 422 contigs and an N50 of 61.5 Mbp (Supplementary Material S2).

We assessed the completeness of the assembly based on a BUSCO analysis of single-copy orthologs v5.1.2 (BUSCO, RRID:SCR_015008) [53, 54] with default parameters and the sauropsid dataset (sauropsida_odb10) in the genome mode.

#### Hi-C sequencing and genome scaffolding

The Hi-C library was constructed with a 250-µL whole-blood sample that was first fixed with 1% formaldehyde for 15 minutes at room temperature. Then, solid glycine powder was added to obtain a final concentration of 125 mM and incubated for 15 minutes at room temperature with periodic mixing. After centrifugation, the pellet was resuspended in phosphate-buffered saline (PBS) + 1% Triton-X solution and incubated at room temperature for 15 minutes. Then, the nuclei were collected after the mixture was spun down. The cross-linked sample was sent on dry ice to Phase Genomics (Seattle, WA, USA) for sequencing. The Hi-C library was generated using the Phase Genomics Proximo Animal kit version 4.0. Briefly, the DNA sample was digested with DpnII and the 5′-overhangs were filled while incorporating a biotinylated nucleotide. The blunt-end fragments were ligated, sheared, and the biotinylated ligation junctions captured with streptavidin beads. The resulting fragments were sequenced on a NovaSeq 6000 (Illumina NovaSeq 6000 Sequencing System, RRID:SCR_020150) 150-bp paired-end run. A total of 680 million reads were produced, corresponding to approximately 85× coverage of the genome (NCBI SRA: SRR18673000) (Table 1).

Overall, 90.3% of the Hi-C reads were aligned to the draft genome assembly, sorted, and merged. Then duplicates were removed using Juicer v1.6 (Juicer, RRID:SCR_017226) [55, 56] with default parameters. Approximately 87% of the reads were found to have Hi-C contacts. Afterward, the 3D-DNA pipeline was run with default parameters (-i 15000, -r 2) to generate a candidate assembly [57, 58], which was reviewed using JBAT v2.10.01 [59]. Finally, a high-quality chromosome-level genome assembly was generated after a visual review on JBAT [59]. A total of 26 pseudo-chromosomes were anchored, corresponding to 97.6% of the estimated genome size, yielding a chromosome-level assembled reference genome with an N50 of 148.6 Mbp (Table 1, Fig. 1C) and a BUSCO completeness of 97.3% (Fig. 1D, Table 1). Genome assembly statistics were visualized with a snail plot in BlobToolKit v2.6.4 [60, 61] (Fig. 1E). The chromosome-level assembly of *A. gigantea* (AldGig_1.0) has the longest contig and scaffold N50 and one of the highest BUSCO completeness scores of all available chromosome-level assembled chelonian genomes (Table 2).

**Table 2:** Contiguity and completeness statistics of all available chromosome-level assembled chelonian genomes.

Species name (Accession No.)	Family	Genome size (Gbp)	Contig N50 (Mbp)	Scaffold N50 (Mbp)	BUSCO completeness*
*Aldabrachelys gigantea* (this study)	Testudinidae	2.374	61.5	148.6	97.3% [S: 96.2%, D: 1.1%], F: 0.4%, M: 2.3%
*Gopherus evgoodei* (GCF_007399415.2)	Testudinidae	2.299	13.027	147.4	97% [S: 95.9, D: 1.1%], F: 0.5%, M: 2.5%
*Chelonia mydas* (GCF_015237465.2)	Cheloniidae	2.134	39.416	134.4	97.3% [S: 96.3%, D: 1.0%], F: 0.4%, M: 2.3%
*Dermochelys coriacea* (GCF_009764565.3)	Dermochelyidae	2.165	7.03	137.6	96.3% [S: 95.3%, D: 1.0%], F: 0.6%, M: 3.1%
*Chrysemys picta bellii* (GCF_000241765.4)	Emydidae	2.481	0.021	16	96.6% [S: 95.7%, D: 0.9%], F: 1.1%, M: 2.3%
*Trachemys scripta elegans* (GCF_013100865.1)	Emydidae	2.126	0.205	140.4	95.0% [S: 94.0%, D: 1.0%], F: 1.2%, M: 3.8%
*Mauremys mutica* (GCF_020497125.1)	Geoemydidae	2.484	15.011	135	97.3%[S: 95.2%, D: 2.1%], F: 0.5%, M: 2.2%
*Mauremys reevesii* (GCF_016161935.1)	Geoemydidae	2.368	33.353	130.5	97.5% [S: 95.9%, D: 1.6%], F: 0.4%, M: 2.1%
*Rafetus swinhoei* (GCA_019425775.1)	Trionychidae	2.238	30.964	132	96.4% [S: 95.3%, D: 1.1%], F: 0.6%, M: 3.0%

* BUSCO score generated from the sauropsid (sauropsida_odb10) database. BUSCO statistics: C, complete; D, duplicated; F, fragmented; M, missing; S, single copy.

#### Repetitive element analysis

To identify, classify, and mask repetitive elements in the *A. gigantea* genome, we first generated a species-specific *de novo* repeat library using RepeatModeler v2.0.1 (RepeatModeler, RRID:SCR_015027) [62, 63]. RepeatModeler utilizes RECON (RECON, RRID:SCR_021170) [64], RepeatScout (RepeatScout, RRID:SCR_014653) [65], and Tandem Repeats Finder (Tandem Repeats Database, RRID:SCR_005659) [66] to detect repeat families *de novo*, to identify and classify consensus sequences. These consensus sequences were then used to softmask the genome with RepeatMasker v4.1.0 (RepeatMasker, RRID:SCR_012954) [67] (-nolow, -xsmall). As a result, 46.7% of the genome (1,114,704,617 bp) were detected as repetitive and softmasked. Long interspersed nuclear elements (LINEs) were identified as the most abundant class of repetitive elements (12.36%), followed by long terminal repeat (LTR) elements (5.78%) (Supplementary Materials S3). The repeat content of the *A. gigantea* genome was found to be slightly higher than the repeat contents of the green sea turtle (*Chelonia mydas*) (41.67%), Goode's thornscrub tortoise (*Gopherus evgoodei*) (41.67%), painted turtle (*Chrysemys picta belli*) (42%), and red-eared slider (*Trachemys scripta elegans*) (45%) genomes [68].

#### RNA extraction and sequencing

A whole-blood sample of approximately 1 mL was collected from an individual named Grosser Bub (“Big Boy”) during routine veterinary blood sampling in the Zurich Zoo. A total of 125 µL of whole blood was immediately diluted with the same amount of water, added into TRIzol™ LS Reagent (Invitrogen, Carlsbad, CA, USA), and stored on ice for <2 hours until extraction. RNA was extracted at the Genetic Diversity Center, ETH, following a combination of a TRIzol™ LS (Invitrogen) RNA isolation protocol and the RNeasy Mini Kit (Qiagen). First, the sample was incubated at room temperature for 5 minutes. Then, 0.2 mL chloroform was added to the sample and the mixture was inverted for 15 seconds, followed by a 3-minute incubation at room temperature. The resulting mixture was centrifuged at 11,000 rpm for 15 minutes at 4°C. After centrifugation, the upper phase containing the RNA was collected, mixed with 1× 70% ethanol, and transferred to an RNeasy spin column. For the remaining procedure, the protocol “Purification of Total RNA from Animal Tissues” of the kit was followed, starting from step 6. Briefly, the RNA was bound to the spin column, washed, and eluted in 30 μL molecular grade water. The initial quality control of the RNA was done on a TapeStation (Agilent) and the concentration was measured with a Qubit Fluorometer (Thermo).

The PacBio IsoSeq library for RNA sequencing (RNA-seq) was produced at the Functional Genomic Center Zurich using the SMRTbell Express Template Prep Kit 2.0 (Pacific Biosciences), according to the manufacturer's instructions. A total of 300 ng RNA was used as input for the cDNA synthesis, which was carried out using the NEBNext® Single Cell/Low Input cDNA Synthesis & Amplification Module (NEB, Ipswich, Massachusetts, United States) and Iso-Seq Express Oligo Kit (Pacific Biosciences) following instructions. To enrich for longer transcripts (>3 Kb), 82 µL ProNex Beads was used for the cleanup of the amplified DNA, as outlined in the protocol. For all subsequent quality control steps, a Bioanalyzer 2100 12-Kb DNA Chip assay (Agilent) and a Qubit Fluorometer (Thermo) were used to assess the size and concentration of the library. The SMRT bell-Polymerase Complex was prepared using the Sequel Binding Kit 3.0 (Pacific Biosciences) and sequenced on a PacBio Sequel instrument using the Sequel Sequencing Kit 3.0 (Pacific Biosciences). In total, one Sequel™ SMRT® Cell 1 M v3 (Pacific Biosciences) was run with one movie of 20 hours per cell, producing ∼1.1 Gbp of HiFi data (NCBI SRA: SRR18674283) (Table 1).

#### Gene prediction and annotation

Gene prediction was performed using a combination of *ab initio* and evidence-based prediction methods (RNA-seq and homology based) with the braker2 pipeline v2.1.5 (BRAKER, RRID:SCR_018964) [69–73]. All gene predictions were performed with pretrained parameter sets for chicken (*Gallus gallus domesticus*), which is the evolutionarily closest taxon for *A. gigantea* available within the software. Using pretrained parameters yielded more complete annotations compared to training with extrinsic evidence (i.e., RNA-seq and protein data) as assessed by BUSCO protein completeness analyses. The *ab initio* prediction was performed by utilizing the softmasked reference genome ​​(–AUGUSTUS_ab_initio –softmasking). Evidence for the transcriptome-based prediction was based on combining information from *A. gigantea* PacBio Iso-seq and all available RNA-seq databases from chelonians in closely related genera (*Chelonoidis* spp. and *Gopherus* spp.; Supplementary Material S4). For the alignment of short- and long-read transcripts, the splice-aware alignment tools STAR v2.7.9 (STAR, RRID:SCR_004463) [74, 75] and minimap2 v2.24 (Minimap2, RRID:SCR_018550) [76, 77] (-ax splice:hq -uf) were used, respectively. Additionally, evidence for the homology-based prediction consisted of a protein database combining all vertebrate proteins in the OrthoDB v10 (OrthoDB, RRID:SCR_011980) [78] and the protein sequences of *G. evgoodei* (NCBI RefSeq: GCF_007399415.2) and *C. n. abingdonii* (NCBI RefSeq: GCF_003597395.1). This dataset was aligned against the chromosome-level assembled reference genome via the ProtHint pipeline v2.6.0 (ProtHint, RRID:SCR_021167) [79, 80]. RNA-seq and homology-based evidence were incorporated for the braker2 pipeline (BRAKER, RRID:SCR_018964) run in –etpmode [73, 79, 81–85]. All gene models derived from *ab initio* and evidence-based methods were integrated into a high-confidence nonredundant gene set by using TSEBRA v1.0.3 [86, 87], with the “keep ab_initio” configuration set. The translated protein sequences from the predicted gene models were searched against protein profiles corresponding to major clades/families of transposon open reading frames by TransposonPSI v1.0 [88]. Overall, 331 genes were identified as likely derived from transposable elements and excluded from the annotation. The resulting gene model set consisted of 23,953 protein-coding genes with a mean gene length of 39,458 bp (including introns) and an average of nine exons per coding sequence (Table 1). The mean gene length is smaller compared to genes of other turtles such as *G. evgoodei* (48 Kbp; NCBI RefSeq: GCF_007399415.2), *C. n. abingdonii* (45 Kbp; NCBI RefSeq: GCF_003597395.1), and *C. mydas* (47 Kbp; NCBI RefSeq: GCF_015237465.2).

The completeness of the annotation was assessed based on single-copy orthologs via BUSCO v5.1.2 (BUSCO, RRID:SCR_015008) [53, 54] with default parameters in the protein mode. The proteome BUSCO completeness scores were 93.7% and 91.9% for the vertebrate (vertebrata_odb10) and sauropsida (sauropsida_odb10) datasets, respectively. The level of BUSCO completeness for the datasets is comparable to those of the annotations of the *C. n. abingdonii* (vertebrata, 96.9%; sauropsida, 97.7%) and *G. evgoodei* (vertebrata, 99.7%; sauropsida, 99.3%) (Supplementary Material S5).

Functional annotation of the encoded proteins was performed using the suite of search tools included in InterProScan v5.53–87.0 (InterProScan, RRID:SCR_005829) [89, 90], with default parameters, in combination with putative gene names derived from UniProtKB/Swiss-prot (UniProtKB/Swiss-Prot, RRID:SCR_021164) [91]. AGAT v0.8.0 [92, 93] was used for summarizing the properties of the structural annotation and for combining the structural and functional annotation results. Of all the prediction gene models, 94.1% could be functionally annotated (Table 1, Supplementary Material S6).

#### Identification of noncoding RNA genes

Transfer RNA (tRNA), ribosomal RNA (rRNA), small nuclear RNA (snRNA), and microRNA (miRNA) were annotated using Infernal v1.1.4 (Infernal, RRID:SCR_011809) [94, 95], which builds covariance models as consensus RNA secondary structure profiles from the genome. The tool then uses the models to search Rfam (Rfam, RRID:SCR_007891) [96], a database of noncoding RNA families. Overall, the homology-based noncoding RNA annotation revealed a total of 6,754 tRNAs, 3,636 rRNAs, 345 snRNAs, and 671 miRNAs encoded in the genome.

#### Mitochondrial genome assembly and evaluation

Mitochondrial reads were extracted from the PacBio HiFi dataset and assembled with Hifiasm v0.15.5 [40, 41] using the MitoHifi v2.0 pipeline [97, 98]. The genome size was 16,467 bp and the assembly was 100% identical at the nucleotide level to the *A. gigantea* mitochondrial reference genome available at NCBI RefSeq with accession number NC_028438.1 [99].

#### Synteny analysis

**Figure 2:**
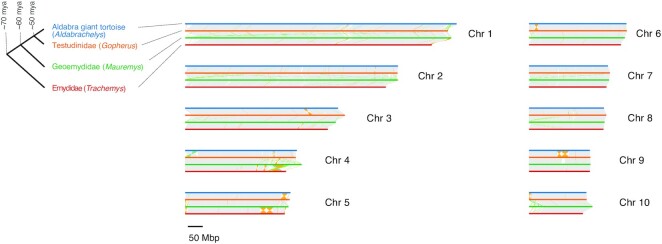
Synteny analysis of 10 chromosomes in *Aldabrachelys gigantea* (blue horizontal lines), *Gopherus evgoodei* (orange horizontal lines), *Mauremys mutica* (green horizontal lines), and *Trachemys scripta elegans* (red horizontal lines) genome assemblies shows high levels of conservation between distantly related chelonian taxa. Gray, yellow, green, and light blue lines between assemblies indicate syntenic regions, inversions, translocations, and duplications, respectively. The four compared assemblies represent all chelonian families (see cladogram on the left, split times from [104]) except Platysternidae within the chelonian superfamily Testudinoidea, which includes families of Emydidae (terrapins), Geoemydidae, and Testudinidae (land-dwelling tortoises). *Trachemys scripta elegans* is from Emydidae, *Mauremys mutica* is from Geoemydidae, and *Gopherus evgoodei* and *Aldabrachelys gigantea* are from Testudinidae.

We investigated the collinearity of *A. gigantea* chromosomes with three other chromosome-level chelonian genome assemblies from three different families, including *G. evgoodei* (Testudinidae; NCBI RefSeq: GCF_007399415.2), the yellow pond turtle (*Mauremys mutica*) (Geoemydidae; NCBI RefSeq: GCF_020497125.1), and *T. s. elegans* (Emydidae; NCBI RefSeq: GCF_013100865.1) (Fig. 2). We analyzed the largest 10 chromosomes corresponding to 75% of the *A. gigantea* assembly. Chromosomes from each genome were aligned to other genomes using minimap2 v2.24 (Minimap2, RRID:SCR_018550) [76, 77] with default parameters (-ax asm5). The resulting alignments were processed with SyRI v1.5.4 [100, 101] to identify syntenic regions and structural rearrangements. The syntenic regions and structural rearrangements for the four chelonian genomes were visualized with plotsr v0.5.3 [102, 103]. Among genomes, we found 1.5 to 1.6 Gbp of syntenic regions and 15.3 to 54.6 Mbp of rearrangements corresponding to 89–94% and 0.8–3% of the compared genome portions, respectively. The rearrangements included 0.2 to 1.4 Mbp of duplications, 2.5 to 7.7 Mbp of translocations, and 6.6 to 51.6 Mbp of inversions. The high ratio of syntenic regions that we found is between chelonian taxa that diverged around 50 to 70 million years ago (mya) [104] (Fig. 2) and is in agreement with previous studies, where the base substitution rate (evolutionary rate) of chelonians was found to be relatively low ([105, 106]; see [107]).

We also performed a complementary collinearity analysis based on orthologous gene sets of *A. gigantea* and the phylogenetically closest available chromosome-level assembled *G. evgoodei* (NCBI RefSeq: GCF_007399415.2) reference genomes (split time ca. 50 mya [104]). We first created orthogroups with the proteomes of the two species using Orthofinder v2.5.4 (OrthoFinder, RRID:SCR_017118) [108, 109]. A total of 41,979 genes (91.3% of total) were assigned to 15,662 orthogroups. The orthologues were then fed in i-ADHoRe v.3.0 [110, 111] to detect genomic regions with statistically significant conserved gene content requiring a minimum of three anchor points within each syntenic region (gap_size = 15, cluster_gap = 30, q_value = 0.05, prob_cutoff = 0.01, anchor_points = 3, alignment_method = gg2, level_2_only = true). Finally, longer-term ancestral synteny detected for the two species was visualized with Circos v0.69–8 (Circos, RRID:SCR_011798) [112, 113] (Supplementary Material S7). Both synteny analysis approaches were providing a consistent picture of high collinearity.

#### Sample collection for low-coverage whole-genome resequencing

**Figure 3:**
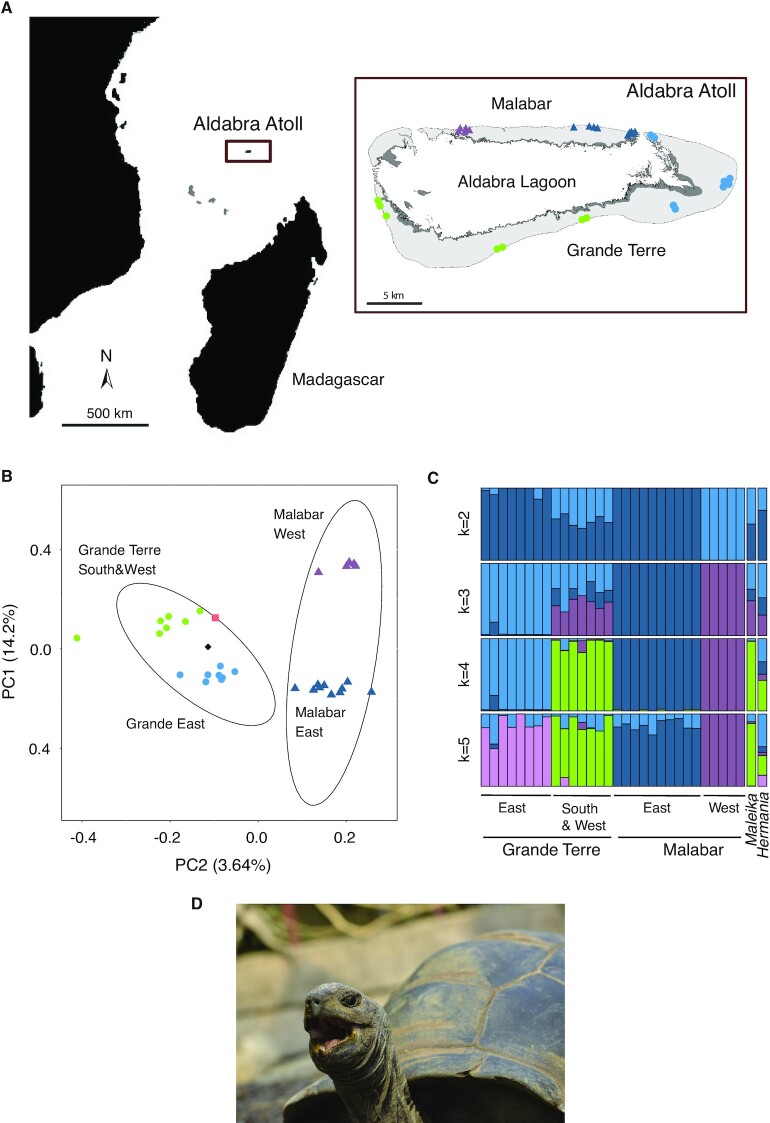
(A) The location of the Aldabra Atoll in the Western Indian Ocean and sampling locations of 30 individuals across the atoll. Every colored mark on the map represents a sampled tortoise. (B) Principal component analysis (PCA) plot of 30 *Aldabrachelys gigantea* individuals from the islands of Grande Terre (East, light blue circles; South & West, green circles) and Malabar (East, dark blue triangles; West, purple triangles) and two individuals from the Zurich Zoo (Hermania, black diamond; Maleika, light pink square). Principal components 1 and 2 account for 14.2% and 3.64% of the overall genetic variability, respectively. (C) Admixture proportions of all the individuals for ancestral populations (*k*) varied from 2 to 5. Each bar represents one individual and shows its admixture proportions. Colors used for *k* = 4 correspond to the colors used in the PCA in panel B. (D) Picture of zoo-housed individual Hermania, originating from the Aldabra Atoll and whose sample contributed to this genome assembly. Photo courtesy of Leyla Davis, Zoo Zürich.

The native distribution of *A. gigantea* is restricted to Aldabra Atoll (Fig. 1B) with deep water channels separating the four main islands (Grande Terre, Malabar, Polymnie, and Picard; Fig. 3A). The smallest island, Polymnie, no longer harbors any tortoises [14]. Tortoises were also harvested to extinction on Picard in the 1800s, but the island has since been repopulated through translocations from Malabar and Grande Terre [114]. In addition to the Atoll, there is an unknown but large number of *ex situ* individuals in zoo, seminatural, or rewilded populations [19]. Assessments of the genetic health of native and rewilded populations will be crucial to inform future species management. However, the uncertainty about genomic vulnerabilities and which *ex situ*individuals to use for rewilding efforts constitute significant barriers.

To assess the utility of our reference genome resources to improve genomic monitoring and inform rewilding efforts, we performed low-coverage whole-genome sequencing of a representative sample of two main islands as well as zoo-housed individuals. Low-coverage sequencing is a powerful and cost-effective approach for conservation and population genomics [115], as well as ancient DNA analyses [116]. We collected blood samples from a total of 30 adult *A. gigantea* (Supplementary Material S8) from Malabar (East, *n* = 10; West, *n* = 5) and Grande Terre (East, *n* = 8; South, *n* = 4; West, *n* = 3) (Fig. 3A). The collection yielded ∼200 µL of blood from the cephalic vein of a front limb. We received a research permit from the Seychelles Bureau of Standards (ref #A0347) for our collection. An export permit was issued by the Ministry of Agriculture, Climate Change and Environment, Republic of Seychelles (permit #A1457), and an import permit was granted by the Federal Food Safety and Veterinary Office of Switzerland to the Department of Evolutionary Biology and Environmental Studies, University of Zurich (permit #19DB000064/22-AS). European zoological institutions currently host over 360 *A. gigantea* individuals [117]. Here, we analyzed two female individuals living in Zurich Zoo, Switzerland. The individual named Hermania was used to create the reference genome, and the individual named Maleika arrived at Zurich Zoo in 1984 and lived there until her death in 2018. The historic information surrounding the exact importation location from Aldabra is sparse or unknown. Sampling from Hermania was performed as described above, and sampling from Maleika was performed by using ∼500 mg of muscle tissue sampled after veterinary necropsy and stored in absolute ethanol until DNA extraction.

#### DNA extraction and sequencing

DNA extraction was performed with 3 µL of blood from Hermania and 15 mg of muscle tissue from Maleika, using the sbeadex™ kit (LGC Genomics, Middlesex, UK), following the manufacturer's protocol for DNA extraction from nucleated red blood cells and tissue, respectively. Genomic DNA concentrations were measured with a dsDNA Broad Range Assay Kit (Qubit 2.0 Fluorometer; Invitrogen). More than 200 ng DNA per sample was sent to Novogene Company (Cambridge, UK) for library preparation and sequencing. Briefly, the genomic DNA was randomly fragmented to a size of 350 bp, end-polished, A-tailed, and ligated with Illumina adapters for Illumina sequencing. After polymerase chain reaction (PCR) enrichment, products were purified (AMPure XP system) and checked for quality on an Agilent 2100 Bioanalyzer (Agilent). Molarity was assessed using real-time PCR. Libraries were sequenced on the Illumina Novaseq 6000 platform with paired-end runs of 150 bp read length. For each of the 32 samples, ∼2.6 Gbp raw reads were generated (NCBI SRA: SRR18674070-101) (Supplementary Material S8, Table 1).

#### Data filtering, alignment, and genotype likelihood estimation

To account for the low-coverage sequencing approach, we assessed genotype likelihoods using the Atlas Pipeline [118, 119]. We first used the GAIA workflow to remove Illumina adapters with TrimGalore v0.6.6 (Trim Galore, RRID:SCR_011847) [120] with default parameters. Only reads longer than 30 bp were retained. Then, reads were aligned to the reference genome with BWA using BWA-MEM v0.7.17 [50] filtering for mapping quality scores >20. Alignments were processed with the RHEA workflow for indel realignment with GATK v3.8 (GATK, RRID:SCR_001876) [121]. A target interval set was created with a representative set of 15 samples, and each individual was realigned together with a representative set of individuals (guidance samples) to enable realignment of low-coverage samples without jointly realigning all samples. The average read depth per sample was 1.62 to 2.06 with a mean of 1.79 (Supplementary Material S8).

We used ANGSD v0.93 (ANGSD, RRID:SCR_021865) [122, 123] to produce genotype likelihoods appropriate for the low coverage of individual samples. GATK was used (GATK, RRID:SCR_001876) [121] to infer major and minor alleles from the likelihoods (doMajorMinor 1, doMaf 1). Quality filtering for the subsequent downstream analyses was performed as follows: only properly paired (only_proper_pairs 1) and unique reads (uniquieOnly 1) were used, and only biallelic sites were retained (skipTrialleleic 1). Nucleotides with base qualities below 20 were discarded. Excessive SNPs around indels and excessive mismatches with the reference were corrected (C50, baq 1, [124]). Sites with read coverage in fewer than 50% of the samples were excluded (minimum representation among samples >50%, -minInd 16). SNPs with a genotype likelihood *P*value <0.001 were retained, producing a final set of 7,131,506 variant sites.

#### Population genetic structure and individual assignments

Our low-coverage sequencing analyses focused on revealing within- and among-island genetic differentiation within the Aldabra population, as well as assigning likely origins for zoo-housed individuals. We first assessed the global genetic structure of the samples using a principal component analysis with PCAngsd v09.85 [125]. Based on a total of 6,651,907 variant sites with a minor allele frequency >0.05, individuals from Malabar and Grande Terre were split into individual groups (Fig.   3B). Both zoo samples were grouped within the group of Grande Terre individuals, revealing the most likely origin for these individuals captured in the 20th century. The principal component analysis also reveals a finer scale east–west population structure within islands confirming recent results based on ddRAD sequencing [126]. We evaluated the impact of more stringently filtering mapping quality (MQ >30 instead of >20), but the resolution of genetic groupings was not meaningfully impacted (Supplementary Material S9).

We also assessed genetic structure using unsupervised Bayesian clustering with NGSAdmix (NGSadmix, RRID:SCR_003208) [127]. We performed pairwise linkage disequilibria (LD) pruning to reduce dependence among SNP loci [127]. Pairwise LD was calculated using ngsLD [128] and LD pruning was performed by allowing a maximum among-SNP distance of 100 Kbp and a minimum weight of 0.5. After LD pruning, 5,862,629 SNPs were retained and 50 replicate runs of NGSAdmix (NGSadmix, RRID:SCR_003208) [127] were performed. We varied the number of clusters (*k*) between 2 and 5 and visualized the assignments with PopHelper v.1.0.10 [129, 130] (Fig. 3C). The admixture analyses for *k* = 2 clusters revealed a main split with groups formed by East Grande Terre together with East Malabar opposed to West Malabar. South and West Grande Terre individuals were assigned to both groups. At *k* = 4, each major sampling region was assigned to a single cluster. The zoo individual Maleika showed a genotype highly consistent with South and West Grande Terre individuals. The individual Hermania (Fig. 3D) was assigned to different Grande Terre regions.

#### Variant annotation

Assessing the genetic health of a species is crucial for its long-term survival, and one major aspect of genetic health is mutation load. For a first glimpse at the distribution of putatively deleterious mutations in the Aldabra giant tortoise genomes, we used SnpEff v5.1 (SnpEff, RRID:SCR_005191) [131, 132] to functionally annotate all SNPs. SnpEff predicts the effects of genetic variants (e.g., loss of function) and allows estimating the expected impact. We identified SNPs in ANGSD v0.93 (ANGSD, RRID:SCR_021865) [122, 123] as described above, but this time including the option -doBCF to create a BCF file. We converted the BCF to a VCF file with BCFtools v1.10.2 (SAMtools/BCFtools, RRID:SCR_005227) [133, 134] applying a minor allele frequency (MAF) filter of ≥0.05. The complete SNP dataset without a MAF filter yielded 7,131,506 SNPs where all SNPs had a minor allele frequency of ≥1%, whereas 6,651,907 SNPs were retained with MAF ≥0.05. We identified 1,077 and 630 SNPs with a putatively high impact on gene function for the complete and MAF ≥0.05 datasets, respectively. For two SNP datasets, we identified 788 and 432 SNPs annotated as loss-of-function variants (e.g., mutated start or stop codons), and 325 and 124 SNPs were identified to have nonsense-mediated decays effect for the complete and MAF ≥0.05 datasets, respectively (see also Supplementary Material S10, S11). Analyzing whether selection is able to remove highly deleterious mutations will provide critical information on the ability of the species to retain high fitness over generations through purging.

## Conclusions

We assembled the first high-quality, chromosome-level annotated genome for the Aldabra giant tortoise, resulting in one of the best-assembled chelonian genomes. Chromosomal collinearity analyses revealed a high degree of conservation even among distantly related tortoise species. We showed that the high-quality resources can be combined with low-coverage resequencing to gain crucial insights into the genetic structure within Aldabra, as well as to resolve the exact origin of zoo-housed individuals. Understanding levels of genomic diversity in both native and *ex situ* populations is crucial to inform rewilding efforts and prioritize conservation efforts. Furthermore, genome-wide analyses of polymorphism can be used to assess the presence of deleterious mutations endangering the long-term health of populations and will allow high-confidence estimates of inbreeding based on runs of homozygosity. Finally, given the exceptionally long life span and large body size of *A. gigantea*, the high-quality genome will inform comparative genomics studies focused on the genetic underpinnings of aging and gigantism.

## Data Availability

The raw sequencing data, the nuclear and mitochondrial genome assemblies, and the annotation produced in this study have been deposited in the NCBI under BioProject accession number PRJNA822095. All supporting data are available in the *GigaScience* GigaDB database [135].

## Editors' Note

A video abstract of this work is available in the *GigaScience* YouTube channel: https://youtu.be/Hak1xO-H8bM

## Additional Files

**Supplementary Material S1**. The *k*-mer (*k* = 17) profile of the *Aldabrachelys gigantea* genome. Consistent with low heterozygosity, most of the *k*-mers form one peak centered on roughly 20× coverage and do not form another peak centered at roughly half the coverage that would represent *k*-mers arising from heterozygous alleles.

**Supplementary Material S2**. Genome contiguity statistics of the assemblies obtained from different assemblers. The column shaded in gray represents our initial assembly obtained via default parameters in Hifiasm.

**Supplementary Material S3**. Summary of repeat annotations.

**Supplementary Material S4**. Accession details of the short-read RNA-seq samples used in this study.

**Supplementary Material S5**. BUSCO statistics for the protein-coding gene annotation of *Aldabrachelys gigantea*, *Chelonoidis abingdonii*, and *Gopherus evgoodei*.

**Supplementary Material S6**. Summary statistics of the functionally annotated protein-coding genes.

**Supplementary Material S7**. Circos plot showing the synteny between the *Aldabrachelys gigantea* Hi-C scaffolds (orange) and *Gopherus evgoodei* assembly pseudo-chromosomes (green).

**Supplementary Material S8**. Details of the location of 30 low-coverage whole-genome resequencing samples.

**Supplementary Material S9**. Principal component analysis plot of 30 wild and two zoo-housed individuals. The analysis was performed with a more stringent mapping quality filter (MQ >30). Principal components 1 and 2 account for 14.5% and 3.77% of the overall genetic variation, respectively. Wild individuals sampled in Grande Terre and Malabar are shown with circles and triangles, respectively (Grande Terre East, light blue; South and West, green; Malabar East, dark blue; West, purple triangles). Two zoo-housed individuals, Hermania and Maleika, are shown with a black diamond and a light pink square, respectively.

**Supplementary Material S10**. Numbers of annotated SNPs with no MAF filtering and MAF ≥0.05 by their impact.

**Supplementary Material S11**. Percentage of effects by their region on the genome (A) effects of SNPs with no MAF filter and (B) MAF ≥0.05.

giac090_GIGA-D-22-00112Revision_1

giac090_GIGA-D-22-00112_Original_Submission

giac090_Response_to_Reviewer_Comments_Original_Submission

giac090_Reviewer_2_Report_Original_Submission VÃctor Quesada -- 6/6/2022 Reviewed

giac090_Reviewer_3_Report_Original_Submission Marc Tollis -- 6/13/2022 Reviewed

giac090_Reviewer_3_Report_Revision_1 Marc Tollis -- 8/2/2022 Reviewed

giac090_Supplemental_Files

## Abbreviations

μg: microgram; μL: microliter; °C: degree Celsius; AGAT: Another Gtf/Gff Analysis Toolkit; ANGSD: Analysis of Next Generation Sequencing Data; baq: base alignment quality; bp: base pairs; BUSCO: Benchmarking Universal Single-Copy Orthologs; BWA: Burrows–Wheeler Aligner; cDNA: complementary DNA; dsDNA: double-strand DNA; EAZA: European Association of Zoos and Aquaria; ETH: Swiss Federal Institute of Technology in Zürich; GAIA: Genome-wide Alignment Including Adapter-trimming; GATK: Genome Analysis Toolkit; Gbp: gigabase pairs; GC: guanine and cytosine; GCE: Genomic Character Estimator; gDNA: genomic DNA; Hi-C: chromosome conformation capture; HiFi: high-fidelity; HMW: high molecular weight; IsoSeq: isoform sequencing; IUCN: International Union for Conservation of Nature; JBAT: Juicebox Assembly Tools; KAT: *k*-mer analysis toolkit; LS: liquid sample; MAF: minor allele frequency; Mbp: megabase pairs; mg: milligram; miRNA: microRNA; mL: milliliter; mM: millimolar; mya: million years ago; NCBI: National Center for Biotechnology Information; NEB: New England Biolabs; ng: nanogram; NGSAdmix: Next Generation Sequencing Admixture; ngsLD: Next Generation Sequencing Linkage Disequilibrium; OrthoDB: orthologous database; PacBio: Pacific Biosciences; PBS: phosphate-buffered saline; PCA: principal component analysis; PCR: polymerase chain reaction; QUAST: Quality Assessment Tool; RefSeq: reference sequence; Rfam: RNA families; RNA-seq: RNA sequencing; rpm: revolutions per minute; rRNA: ribosomal RNA; Sauropsida_odb10: sauropsids orthologous database 10; SMRT: single molecule real time; SNP: single-nucleotide polymorphism; snRNA: small nuclear RNA; SRA: Sequence Read Archive; STAR: Spliced Transcripts Alignment to a Reference; SyRI: Synteny and Rearrangement Identifier; tRNA: transfer RNA; TSEBRA: Transcript Selector for BRAKER; UniProtKB: Universal Protein Knowledgebase; Vertebrata_odb10: vertebrate orthologous database 10.

## Competing Interests

The authors declare that they have no competing interests.

## Funding

This study was funded through the Research Talent Development Fund of the University of Zürich, the Swiss National Science Foundation (Project No. 31003A_182343), and University of Zurich Internal Funds, all of which were given to C.G.

## Authors' Contributions

F.G.Ç. and C.G. conceived the study design. F.G.Ç. carried out all DNA and RNA extractions and bioinformatic analyses with guidance from D.C. and C.G. D.H. and L.D. coordinated the sampling of the zoo animals. N.B. provided administrative support for sampling on Aldabra Atoll and L.A. managed the collection, storage, and transport of samples from wild individuals. F.G.Ç. wrote the manuscript with guidance from C.G. and substantial input from D.C. All authors revised the manuscript.
