# Supplementary material for: Chromosome-level genome assembly for the Aldabra giant tortoise enables insights into the genetic health of a threatened population
Source: Gigascience. 2022 Oct 12;11:giac090. doi: 10.1093/gigascience/giac090 (PMC9553416; doi:10.1093/gigascience/giac090)
Supplement: giac090_GIGA-D-22-00112_Original_Submission [file giac090_giga-d-22-00112_original_submission.pdf]

GigaScience

Chromosome-level genome assembly for the Aldabra giant tortoise enables insights into the genetic health of a threatened population

--Manuscript Draft--

**Manuscript Number:**	GIGA-D-22-00112
**Full Title:**	Chromosome-level genome assembly for the Aldabra giant tortoise enables insights into the genetic health of a threatened population
**Article Type:**	Data Note
**Funding Information:**	Research Talent Development Fund of the University of Zürich — Dr. Christine Grossen<br>Schweizerischer Nationalfonds zur Förderung der Wissenschaftlichen Forschung (31003A_182343) — Dr. Christine Grossen<br>University of Zurich Internal Funds — Dr. Christine Grossen

Abstract:

Background

The Aldabra giant tortoise (*Aldabrachelys gigantea*) is one of only two giant tortoise species left in the world. The species is endemic to Aldabra Atoll in Seychelles and is considered vulnerable due to its limited distribution and threats posed by climate change. Genomic resources for *A. gigantea* are lacking, hampering conservation efforts focused on both wild and ex-situ populations. A high-quality genome would also open avenues to investigate the genetic basis of the exceptionally long lifespan.

Findings

We produced the first chromosome-level *de novo* genome assembly of *A. gigantea* using PacBio High-Fidelity sequencing and high-throughput chromosome conformation capture (Hi-C). We produced a 2.37 Gbp assembly with a scaffold N50 of 148.6 Mbp and a resolution into 26 chromosomes. RNAseq-assisted gene model prediction identified 23,953 protein-coding genes and 1.1 Gbp of repetitive sequences. Synteny analyses among turtle genomes revealed high levels of chromosomal collinearity even among distantly related taxa. To assess the utility of the high-quality assembly for species conservation, we performed a low-coverage re-sequencing of 30 individuals from wild populations and two zoo individuals. Our genome-wide population structure analyses detected genetic population structure in the wild and identified the most likely origin of the zoo-housed individuals. We further identified putatively deleterious mutations to be monitored.

Conclusions

We establish a high-quality chromosome-level reference genome for *A. gigantea* , and one of the most complete turtle genomes available. We show that low-coverage whole-genome resequencing, for which alignment to the reference genome is a necessity, is a powerful tool to assess the population structure in the wild population and reveal the geographic origins of ex-situ individuals relevant for genetic diversity management and rewilding efforts.

**Corresponding Author:**	F. Gözde Çilingir<br>University of Zurich<br>Zurich, SWITZERLAND
**Corresponding Author Secondary Information:**	
**Corresponding Author's Institution:**	University of Zurich
**Corresponding Author's Secondary Institution:**	
**First Author:**	F. Gözde Çilingir

Powered by Editorial Manager® and ProduXion Manager® from Aries Systems Corporation

First Author Secondary Information:	
Order of Authors:	F. Gözde Çilingir
	Luke A'bear
	Dennis Hansen
	Leyla R. Davis
	Nancy Bunbury
	Arpat Ozgul
	Daniel Croll
	Christine Grossen
Order of Authors Secondary Information:	
Additional Information:	
Question	Response
Are you submitting this manuscript to a special series or article collection?	No
**Experimental design and statistics**<br><br>Full details of the experimental design and statistical methods used should be given in the Methods section, as detailed in our Minimum Standards Reporting Checklist. Information essential to interpreting the data presented should be made available in the figure legends.<br><br>Have you included all the information requested in your manuscript?	Yes
**Resources**<br><br>A description of all resources used, including antibodies, cell lines, animals and software tools, with enough information to allow them to be uniquely identified, should be included in the Methods section. Authors are strongly encouraged to cite Research Resource Identifiers (RRIDs) for antibodies, model organisms and tools, where possible.<br><br>Have you included the information requested as detailed in our Minimum Standards Reporting Checklist?	Yes

**Availability of data and materials**<br><br>All datasets and code on which the conclusions of the paper rely must be either included in your submission or deposited in publicly available repositories (where available and ethically appropriate), referencing such data using a unique identifier in the references and in the "Availability of Data and Materials" section of your manuscript.<br><br>Have you have met the above requirement as detailed in our Minimum Standards Reporting Checklist?	Yes

Manuscript Click here to access/download;Manuscript;Main_text.docx

Chromosome-level genome assembly for the Aldabra giant tortoise enables insights into the genetic health of a threatened population

Çilingir, F.G.[1*], *A'Bear, L.*[2], *Hansen, D.*[3,4], *Davis, L.R.*[5], *Bunbury, N.*[2,6], *Ozgul, A.*[1], *Croll, D.*[7#,*], *Grossen, C.*[1#,*]

1 Department of Evolutionary Biology and Environmental Studies, University of Zurich, 8057 Zurich, Switzerland

2 Seychelles Islands Foundation, PO Box 853, Victoria, Republic of Seychelles

3 Zoological Museum, University of Zurich, Karl-Schmid-Strasse 4, 8006 Zurich, Switzerland

4 Indian Ocean Tortoise Alliance, Ile Cerf, Victoria, Republic of Seychelles

5 Zoo Zürich, Zürichbergstrasse 221, 8044 Zurich, Switzerland

6 Centre for Ecology and Conservation, College of Life and Environmental Sciences, University of Exeter, Penryn, Cornwall, TR10 9FE, UK.

7 Institute of Biology, University of Neuchâtel, 2000 Neuchâtel, Switzerland

[*]Corresponding authors: fgcilingir@gmail.com, christine.grossen@gmail.com, daniel.croll@unine.ch

[#]These authors have contributed equally to this work.

Abstract

Background

The Aldabra giant tortoise (*Aldabrachelys gigantea*) is one of only two giant tortoise species left in the world. The species is endemic to Aldabra Atoll in Seychelles and is considered vulnerable due to its limited distribution and threats posed by climate change. Genomic resources for *A. gigantea* are lacking, hampering conservation efforts focused on both wild and ex-situ populations. A high-quality genome would also open avenues to investigate the genetic basis of the exceptionally long lifespan.

Findings

We produced the first chromosome-level *de novo* genome assembly of *A. gigantea* using PacBio High-Fidelity sequencing and high-throughput chromosome conformation capture (Hi-C). We produced a 2.37 Gbp assembly with a scaffold N50 of 148.6 Mbp and a resolution into 26 chromosomes. RNAseq-assisted gene model prediction identified 23,953 protein-coding genes and 1.1 Gbp of repetitive sequences. Synteny analyses among turtle genomes revealed high levels of chromosomal collinearity even among distantly related taxa. To assess the utility of the high-quality assembly for species conservation, we performed a low-coverage re-sequencing of 30 individuals from wild populations and two zoo individuals. Our genome-wide population structure analyses detected genetic population structure in the wild and identified the most likely origin of the zoo-housed individuals. We further identified putatively deleterious mutations to be monitored.

Conclusions

We establish a high-quality chromosome-level reference genome for *A. gigantea*, and one of the most complete turtle genomes available. We show that low-coverage whole-genome

resequencing, for which alignment to the reference genome is a necessity, is a powerful tool to assess the population structure in the wild population and reveal the geographic origins of ex-situ individuals relevant for genetic diversity management and rewilding efforts.

Keywords: Aldabrachelys gigantea, genome assembly, HiFi sequencing, Hi-C sequencing

Background

As human activities drive our planet into the sixth mass extinction[1], genomic technologies are becoming an increasingly important tool for conservation researchers. The establishment of reference-quality genomes for species of conservation concern makes key contributions to the study of common genetic health issues elucidating the full spectrum of genomic diversity, accurately quantifying inbreeding, mutation load, and introgression, detecting hybridization, and identifying adaptive variation in the face of rapidly changing environments[2]. The number of available reference genomes for non-model species has been increasing thanks to ongoing efforts in several global genome consortia, such as the Earth Biogenome Project[3], the Vertebrate Genomes Project[4,5], and the Global Invertebrate Genomics Alliance[6]. However, available reference genomes of non-model species are not homogeneously distributed across the tree of life. Only three reference genomes represent the Testudinidae family (or tortoises) from two genera, with two genomes being annotated and only one assembled to chromosome level. Tortoises have been integral components of global ecosystems for about 220 million years[7] contributing to seed dispersal, nutrient and mineral cycling, and carbon storage[8]. Over their long evolutionary history, giant tortoises, in particular, have evolved a life history characterized by delayed maturity, extended reproductive lives, and extreme longevity[9].

Currently, there are only two extant giant tortoise taxa, both of which face extinction threats[10]. Galápagos giant tortoises (*Chelonoidis niger* and subspecies thereof, formerly *Chelonoidis niger* species complex) are native to the Galápagos Islands in the eastern Pacific Ocean, and taxa of this group are listed as either vulnerable, endangered, or extinct according to IUCN Red List (v2.3). Aldabra giant tortoises (*Aldabrachelys gigantea*) (Fig. 1A) are native to Aldabra Atoll in the western Indian Ocean (Fig. 1B). Due to their extremely limited distribution in the wild and the threats posed by climate change, the species is vulnerable. Genomes of giant tortoises may harbor clues to their exceptional life-history traits. Assessing genome-wide variation within species, including deleterious mutation load, will critically improve conservation management programs[11]. The recently established reference genome for one of the Galápagos giant tortoises, *Chelonoidis niger abingdonii*, revealed insights into potentially aging, disease, and cancer-related gene functions by analyzing gene content evolution among tortoises[12]. For Aldabra giant tortoises, only short-read sequencing data is available from the same study[12].

Aldabrachelys gigantea have been successfully used in rewilding projects on several Western Indian Ocean Islands, whose endemic giant tortoise species have recently gone extinct [13]. The introduced populations act as ecological replacements for the extinct species and take a central role in shaping and sustaining large-scale vegetation dynamics as the largest frugi- and herbivore[14–17]. *Aldabrachelys gigantea* have been introduced on the Mauritian islets of Ile Aux Aigrettes and Round Island and on the island of Rodrigues[18]. Monitoring the effectiveness of these rewilding projects will be crucial to catalyzing larger projects in Madagascar[19]. *Aldabrachelys gigantea* rewilding programs require genomic monitoring to reduce founder effects and maximize genetic variation in newly introduced populations[20]. Finally, uncertainties exist about the existence of additional *Aldabrachelys* lineages, as well as

the number and taxonomic status of extinct lineages[10] due to weak morphological resolution and low-resolution genetic marker sets[21,22].

In this study, we present the first high-quality chromosome-level genome of *A. gigantea* using PacBio HiFi sequencing and Hi-C sequencing for scaffolding. We assessed the utility of the reference genome by performing low coverage whole-genome resequencing for a total of 32 tortoises. We inferred the genetic structure of the wild population and the likely origin of zoo-housed individuals.

Data description

Genome sequencing and assembly

DNA extraction, PacBio library preparation, and sequencing

In December 2020, during routine veterinary blood sampling, a subsample of approximately 3 mL of whole blood was collected from a female *A. gigantea* (named Hermania) living in the Zurich Zoo since 1955. Because blood was subsampled during a routine veterinary blood sampling, no additional ethical approval was required. Whole blood was taken from the animal's dorsal tail vein and stored on ice in a heparin-coated blood collection tube. DNA extraction was carried out at the Genetic Diversity Center, ETH, Zurich, according to the manufacturer's instructions of MagAttract® High Molecular Weight DNA (HMW) Kit (Qiagen), with a single modification: instead of using 200 µl whole blood as suggested for blood samples with non-nucleated red blood cells, a total of 50 µl whole blood was used. The purified DNA was eluted in 200 µl molecular-grade water. Subsequent steps, including gDNA quality control, PacBio HiFi library preparation, and sequencing, were carried out at the Functional Genomic Center Zurich, ETH.

The input HMW genomic DNA concentration was measured using a Qubit Fluorometer (Thermo), and the DNA integrity was checked on a Femto Pulse Device (Agilent). The HiFi library preparation started with 14 µg HMW DNA. The PacBio HiFi library was produced using the SMRTbell® Express Template Prep Kit 2.0 (Pacific Biosciences), according to the manufacturer's instructions. Briefly, the DNA sample was mechanically sheared to an average size of 20 Kbp using a Megaruptor 3 Device (Diagenode). A Femto Pulse gDNA analysis assay (Agilent) was used to assess the resulting fragment size distribution. The sheared DNA sample was DNA damage repaired and end-repaired using polishing enzymes. PacBio sequencing adapters were ligated to the DNA template. A Blue Pippin device (Sage Science) was used to size-select fragments >15 Kbp. The size-selected library was quality inspected and quantified using a Femto Pulse gDNA analysis assay (Agilent) and a Qubit Fluorometer (Thermo), respectively. The SMRT® bell-Polymerase Complex was prepared using the Sequel® II Binding Kit 2.0 and Internal Control 1.0 (Pacific Biosciences) and sequenced on a PacBio Sequel II instrument using the Sequel II Sequencing Kit 2.0 (Pacific Biosciences). In total, two Sequel II SMRT Cells 8M (Pacific Biosciences) were run, taking one movie of 30 hours per cell. This yielded 49.4 Gbp of HiFi reads with a mean read length of 22.8 Kbp, which corresponds to approximately 20.8× coverage of the genome (NCBI SRA: SRR18672579) (Table 1).

Nuclear genome assembly, contamination scan, and evaluation

The consensus circular sequences per each Sequel II SMRT Cell (Pacific Biosciences) were filtered for adapter contamination with HiFiAdapterFilt v2.0.0 [23,24]. Overall, 0.008% of the HiFi reads were filtered out. Genome size and heterozygosity rate were estimated based on the 17-mer frequency of the cleaned HiFi reads with GCE v1.0.2 (GCE, RRID: SCR_017332) [25,26]. Our results indicate that *A. gigantea* has an estimated genome size of 2.37 Gbp

(Supplementary Material S1) and low heterozygosity of 0.072% (corresponding to 0.72 SNPs per 1 Kbp).

The reads were then assembled with the default parameters of HiCanu v2.1.1 (Canu, RRID:SCR_015880)[27,28], Improved Phased Assembler v1.3.2 (IPA HiFi Genome Assembler, RRID:SCR_021966) [29], and Hifiasm v0.15.5 (Hifiasm, RRID:SCR_021069)[30,31]. Additionally, an option for assembling inbred/homozygous genomes (-l 0) within Hifiasm (Hifiasm, RRID:SCR_021069)[30,31] was also tested. Main contiguity statistics were calculated with QUAST v5.0.2 (QUAST, RRID:SCR_001228)[32,33]. The subsequent analyses were performed with the draft assembly obtained via Hifiasm (Hifiasm, RRID:SCR_021069)[30,31] with default parameters because it provided the most contiguous and complete assembly with 483 contigs and an N50 of 61.5 Mbp (Supplementary Material S2).

Scanning for contaminant contigs in the draft assembly was performed by following three approaches. First, the draft assembly was split into 5 Kbp segments using SeqKit v0.16.1 (SeqKit, RRID:SCR_018926)[34,35]. Each segment was blasted against the full NCBI non-redundant protein database by running diamond v2.0.9 (DIAMOND, RRID:SCR_016071)[36,37] with the blastx option. The decision of a segment coming from an external source was given by considering the bitscore evalue and GC content, and none of the hits were considered a significant match with cut-off values of 30, 0.0001, 70%, respectively. Second, we assessed k-mer profiles of the most probable sources of contamination human genome (NCBI RefSeq: GCF_000001405.39) and the *A. gigantea* mitochondrial genome; NCBI RefSeq: NC_028438.1), were compared with the k-mer profile of the draft assembly using KAT v2.4.1 (KAT, RRID:SCR_016741)[38,39]). The k-mer profiles were well distinct

suggesting no apparent contamination. Third, the previously published *A. gigantea* whole-genome resequencing dataset (NCBI SRA: SRX4741543)[12] was mapped against our assembly with BWA-MEM v0.7.17 (BWA, RRID:SCR_010910)[40]. The read coverage profile was examined with Qualimap v2.2.1 (QualiMap, RRID:SCR_001209)[41,42]. The resequencing dataset had 27× coverage, therefore we discarded contigs from the assembly with less than 10× or more than 100× aligned read depth. All contaminant filtering steps combined, 62 contigs were removed from the assembly, resulting in a final set of 422 contigs and an N50 of 61.5 Mbp (Supplementary Material S2).

We assessed the completeness of the assembly based on a BUSCO analysis of single-copy orthologs v5.1.2 (BUSCO, RRID:SCR_015008)[43,44] with default parameters and the sauropsid dataset (sauropsida_odb10) in the genome mode.

Hi-C sequencing and genome scaffolding

The Hi-C library was constructed with a 250 µl whole blood sample that was first fixed with 1% formaldehyde for 15 min at room temperature. Then, solid glycine powder was added to obtain a final concentration of 125 mM and incubated for 15 min at room temperature with periodic mixing. After centrifugation, the pellet was resuspended in PBS + 1% Triton-X solution and incubated at room temperature for 15 min. Then, the nuclei were collected after the mixture was spun down. The cross-linked sample was sent on dry ice to Phase Genomics (Seattle, Washington, USA) for sequencing. The Hi-C library was generated using the Phase Genomics Proximo Animal kit version 4.0. Briefly, the DNA sample was digested with DpnII and the 5'-overhangs were filled while incorporating a biotinylated nucleotide. The blunt-end fragments were ligated, sheared, and the biotinylated ligation junctions captured with streptavidin beads. The resulting fragments were sequenced on a NovaSeq 6000 150 bp paired-

end run. A total of 680 million reads were produced, corresponding to approximately 85× coverage of the genome (NCBI SRA: SRR18673000) (Table 1).

Overall, 90.3% of the Hi-C reads were aligned to the draft genome assembly, sorted, and merged. Then duplicates were removed using Juicer v1.6 (Juicer, RRID:SCR_017226)[45,46] with default parameters. Approximately 87% of the reads were found to have Hi-C contacts. Afterward, the 3D-DNA pipeline was run with default parameters to generate a candidate assembly[47,48], which was reviewed using JBAT v2.10.01[49]. Finally, a high-quality chromosome-level genome assembly was generated after a visual review on JBAT[49]. A total of 26 pseudo-chromosomes were anchored, corresponding to 97.6% of the estimated genome size, yielding a chromosome-level assembled reference genome with an N50 of 148.6 Mbp (Table 1, Fig. 1C) and a BUSCO completeness of 97.3% (Fig. 1D, Table 1). Genome assembly statistics were visualized with a snail plot in BlobToolKit v2.6.4 [50,51] (Fig. 1E). The chromosome level assembly of *A. gigantea* (AldGig_1.0) has the longest contig and scaffold N50, and one of the highest BUSCO completeness scores of all available chromosome-level assembled chelonian genomes (Table 2).

Repetitive element analysis

To identify, classify, and mask repetitive elements in the *A. gigantea* genome, we first generated a species-specific *de novo* repeat library using RepeatModeler v2.0.1 (RepeatModeler, RRID:SCR_015027)[52,53]. RepeatModeler utilizes RECON (RECON, RRID:SCR_021170)[54], RepeatScout (RepeatScout, RRID:SCR_014653)[55], and Tandem Repeats Finder (Tandem Repeats Database, RRID:SCR_005659)[56] to detect repeat families *de novo*, to identify and classify consensus sequences. These consensus sequences were then used to softmask the genome with RepeatMasker v4.1.0 (RepeatMasker,

RRID:SCR_012954)[57]. As a result, 46.7% of the genome (1,114,704,617 bp) were detected as repetitive and softmasked. Long interspersed nuclear elements (LINEs) were identified as the most abundant class of repetitive elements (12.36%) followed by long terminal repeat (LTR) elements (5.78%) (Supplemenary Material S3).

RNA extraction and sequencing

A whole blood sample of approximately 1 mL was collected from an individual named Grosser Bub ('Big Boy') during routine veterinary blood sampling in the Zurich Zoo. A total of 125 µl of whole blood was immediately diluted with the same amount of water, added into TRIzol™ LS Reagent (Invitrogen), and stored on ice for < 2 hours until extraction. RNA was extracted at the Genetic Diversity Center, ETH, following a combination of a TRIzol™ LS (Invitrogen) RNA isolation protocol and the RNeasy Mini Kit (Qiagen). First, the sample was incubated at room temperature for 5 min. Then, 0.2 mL chloroform was added to the sample and the mixture was inverted for 15 seconds, followed by 3 min incubation at room temperature. The resulting mixture was centrifuged at 11,000 rpm for 15 min at 4°C. After centrifugation, the upper phase containing the RNA was collected, mixed with 1x 70% ethanol, and transferred to an RNeasy spin column. For the remaining procedure, the protocol "Purification of Total RNA from Animal Tissues" of the kit was followed, starting from step 6. Briefly, the RNA was bound to the spin column, washed, and eluted in 30 µl molecular grade water. The initial quality control of the RNA was done on a TapeStation (Agilent) and the concentration was measured with a Qubit Fluorometer (Thermo).

The PacBio IsoSeq library for RNA sequencing was produced at the Functional Genomic Center Zurich using the SMRTbell Express Template Prep Kit 2.0 (Pacific Biosciences), according to the manufacturer's instructions. A total of 300 ng RNA was used as input for the

cDNA synthesis, which was carried out using the NEBNext® Single Cell/Low Input cDNA Synthesis & Amplification Module (NEB) and Iso-Seq Express Oligo Kit (Pacific Biosciences) following instructions. To enrich for longer transcripts (>3 kb), 82 µl ProNex Beads were used for the clean-up of the amplified DNA, as outlined in the protocol. For all subsequent quality control steps, a Bioanalyzer 2100 12 Kb DNA Chip assay (Agilent) and a Qubit Fluorometer (Thermo) were used to assess the size and concentration of the library. The SMRT bell-Polymerase Complex was prepared using the Sequel Binding Kit 3.0 (Pacific Biosciences) and sequenced on a PacBio Sequel instrument using the Sequel Sequencing Kit 3.0 (Pacific Biosciences). In total, one Sequel™ SMRT® Cell 1M v3 (Pacific Biosciences) was run with one movie of 20 hours per cell producing ~1.1 Gbp of HiFi data (NCBI SRA: SRR18674283) (Table 1).

Gene prediction and annotation

Gene prediction was performed using a combination of *ab initio* and evidence-based prediction methods (RNA-seq and homology-based) with the braker2 pipeline v2.1.5 (BRAKER, RRID:SCR_018964)[58–62]. All gene predictions were performed with pre-trained parameter sets for chicken, which is the evolutionarily closest taxon for *A. gigantea* available within the software. Using pre-trained parameters yielded more complete annotations compared to training with extrinsic evidence (i.e. RNA-seq and protein data). The *ab initio* prediction was performed by utilizing the soft-masked reference genome. Evidence for the transcriptome-based prediction was based on combining information from *A. gigantea* PacBio Iso-seq and all available RNA-seq databases from chelonians in closely related genera (*Chelonoidis* spp. and *Gopherus* spp.; Supplementary Material S4). For the alignment of short and long read transcripts, the splice-aware alignment tools STAR v2.7.9 (STAR, RRID:SCR_004463)[63,64] and minimap2 v2.24 (Minimap2, RRID:SCR_018550)[65,66]

were used, respectively. Additionally, evidence for the homology-based prediction consisted of a protein database combining all vertebrate proteins in the OrthoDB v10 (OrthoDB, RRID:SCR_011980)[67] and the protein sequences of *Gopherus evgoodei* (NCBI RefSeq: GCF_007399415.2) and *Chelonoidis niger abingdonii* (NCBI RefSeq: GCF_003597395.1). This dataset was aligned against the chromosome-level assembled reference genome via the ProtHint pipeline v2.6.0 (ProtHint, RRID:SCR_021167)[68,69]. RNA-seq and homology-based evidence were incorporated for the braker2 pipeline (BRAKER, RRID:SCR_018964) run in --etpmode[62,68,70–74]. All gene models derived from *ab initio* and evidence-based methods were integrated into a high confidence non-redundant gene set by using TSEBRA v1.0.3 [75,76], with the "keep ab_initio" configuration set. The translated protein sequences from the predicted gene models were searched against protein profiles corresponding to major clades/families of transposon open reading frames by TransposonPSI v1.0 [77]. Overall, 331 genes were identified as likely derived from transposable elements and excluded from the annotation. The resulting gene model set consisted of 23,953 protein-coding genes with a mean gene length of 39,458 bp and an average of nine exons per coding sequence (Table 1).

The completeness of the annotation was assessed based on single-copy orthologs via BUSCO v5.1.2(BUSCO, RRID:SCR_015008)[43,44] with default parameters in the protein mode. The proteome BUSCO completeness scores were 93.7% and 91.9% for the vertebrate (vertebrata_odb10) and sauropsida (sauropsida_odb10) datasets, respectively. The level of BUSCO completeness for the datasets is comparable to those of the annotations of the *C. n. abingdonii* (vertebrata, 96.9%; sauropsida, 97.7%) and *G. evgoodei* (vertebrata, 99.7%; sauropsida, 99.3%) (Supplementary Material S5).

Functional annotation of the encoded proteins was performed using the suite of search tools included in InterProScan v5.53-87.0 (InterProScan, RRID:SCR_005829)[78,79], with default parameters, in combination with putative gene names derived from UniProtKB/Swiss-prot (UniProtKB/Swiss-Prot, RRID:SCR_021164)[80]. AGAT v0.8.0[81,82] was used for summarizing the properties of the structural annotation and for combining the structural and functional annotation results. Of all the prediction gene models, 94.1% could be functionally annotated (Table 1, Supplementary Material S6).

Identification of non-coding RNA genes

tRNA, rRNA, snRNA, and miRNA were annotated using Infernal v1.1.4 (Infernal, RRID:SCR_011809)[83,84], which builds covariance models as consensus RNA secondary structure profiles from the genome. The tool then uses the models to search Rfam (Rfam, RRID:SCR_007891)[85], a database of non-coding RNA families. Overall, the homology-based non-coding RNA annotation revealed a total of 6,754 tRNAs, 3,636 rRNAs, 345 snRNAs, and 671 miRNAs encoded in the genome.

Mitochondrial genome assembly and evaluation

Mitochondrial reads were extracted from the PacBio HiFi dataset and assembled with Hifiasm v0.15.5 (Hifiasm, RRID:SCR_021069)[30,31] using the MitoHifi v2.0 pipeline[86,87]. The genome size was 16,467 bp and the assembly was 100% identical at the nucleotide level to the *A. gigantea* mitochondrial reference genome available at NCBI RefSeq with accession no NC_028438.1[88].

Synteny analysis

We investigated the collinearity of *A. gigantea* chromosomes with three other chromosome-level chelonian genome assemblies from three different families including *Gopherus evgoodei* (Testudinidae; NCBI RefSeq: GCF_007399415.2), *Mauremys mutica* (Geoemydidae; NCBI RefSeq: GCF_020497125.1) and *Trachemys scripta elegans* (Emydidae; NCBI RefSeq: GCF_013100865.1) (Fig. 2). We analyzed the largest 10 chromosomes corresponding to 75% of the *A. gigantea* assembly. Chromosomes from each genome were aligned to other genomes using minimap2 v2.24 (Minimap2, RRID:SCR_018550)[65,66] with default parameters (-ax asm5). The resulting alignments were processed with SyRI v1.5.4 [89,90] to identify syntenic regions and structural rearrangements. The syntenic regions and structural rearrangements for the four chelonian genomes were visualized with plotsr v0.5.3[91,92]. Among genomes, we found between 1.5–1.6 Gbp of syntenic regions and 15.3–54.6 Mbp of rearrangements corresponding to 89–94% and 0.8–3% of the compared genome portions, respectively. The rearrangements included 0.2–1.4 Mbp of duplications, 2.5–7.7 Mbp of translocations, and 6.6–51.6 Mbp of inversions. The high ratio of syntenic regions that we found are between chelonian taxa that diverged around 50–70 mya[93] (Fig. 2) and is in agreement with previous studies, where the base substitution rate (evolutionary rate) of chelonians was found to be relatively low (see[94]).

We also performed a complementary collinearity analysis based on orthologous gene sets of *A. gigantea* and the phylogenetically closest available chromosome-level assembled *Gopherus evgoodei* (NCBI RefSeq: GCF_007399415.2) reference genomes (split time ca. 50 my[93]). We first created orthogroups with the proteomes of the two species using Orthofinder v2.5.4 (OrthoFinder, RRID:SCR_017118)[95,96]. A total of 41979 genes (91.3% of total) were assigned to 15662 orthogroups. The orthologues were then fed in i-ADHoRe v.3.0 [97,98] to detect genomic regions with statistically significant conserved gene content requiring a

minimum of three anchor points within each syntenic region (gap_size=15, cluster_gap=30, q_value=0.05, prob_cutoff=0.01, anchor_points=3, alignment_method=gg2, level_2_only=true). Finally, longer-term ancestral synteny detected for the two species was visualized with Circos v0.69-8 (Circos, RRID:SCR_011798)[99,100] (Supplementary Material S7). Eventually, the high ratio of synteny revealed by our complementary collinnearity analysis supported our findings on the chromosomal synteny analysis.

Sample collection for low coverage whole-genome resequencing

The native distribution of *A. gigantea* is restricted to the Aldabra Atoll (Fig. 1B) with deep water channels separating the four main islands (Grande Terre, Malabar, Polymnie, and Picard; Fig. 3A). The smallest island Polymnie no longer harbors any tortoises[101]. Tortoises were also harvested to extinction on Picard in the 1800s but the island has since been re-populated through translocations from Malabar and Grande Terre[102]. In addition to the Atoll, there is an unknown, but large number of ex-situ individuals in zoo, semi-natural, or rewilded populations [13]. Assessments of the genetic health of native and rewilded populations will be crucial to inform future species management. However, the uncertainty about genomic vulnerabilities and which ex-situ individuals to use for re-wilding efforts constitute significant barriers.

To assess the utility of our reference genome resources to improve the genomic monitoring and inform re-wilding efforts, we performed low-coverage whole-genome sequencing of a representative sample of two main islands as well as zoo-housed individuals. Low-coverage sequencing is a powerful and cost-effective approach for conservation and population genomics [103], as well as ancient DNA analyses [104]. We collected blood samples from a total of 30 adult *A. gigantea* (Supplementary Material S8) from Malabar (East, N=10; West,

N=5) and Grande Terre (East, N=8; South, N=4; West, N=3) (Fig. 3A). The collection yielded ~200 µl of blood from the cephalic vein of a front limb. We received a research permit from the Seychelles Bureau of Standards (ref #A0347) for our collection. European institutions currently host over 360 *A. gigantea* [105]. Here, we analyzed two female individuals living in Zurich Zoo, Switzerland. The individual named Hermania was used to create the reference genome, and the individual named Maleika arrived at Zurich Zoo in 1984 and lived there until her death in 2018. The historic information surrounding the exact importation location from Aldabra is sparse or unknown. Sampling from Hermania was performed as described above, and sampling from Maleika was performed by using ~500 mg of muscle tissue sampled after veterinary necropsy and stored in absolute ethanol until DNA extraction.

DNA extraction, and sequencing

DNA extraction was performed with 3 µl of blood from Hermania and 15 mg of muscle tissue from Maleika, using the sbeadex™ kit (LGC Genomics, Middlesex, UK), following the manufacturer's protocol for DNA extraction from nucleated red blood cells and tissue, respectively. Genomic DNA concentrations were measured with a dsDNA Broad Range Assay Kit (Qubit 2.0 Fluorometer, Invitrogen, Carlsbad). More than 200 ng of DNA per sample was sent to Novogene Company (Cambridge, United Kingdom) for library preparation and sequencing. Briefly, the genomic DNA was randomly fragmented to a size of 350 bp, end-polished, A-tailed and ligated with Illumina adapters of Illumina sequencing. After PCR enrichment, products were purified (AMPure XP system) and checked for quality on an Agilent 2100 Bioanalyzer (Agilent Technologies, CA, USA). Molarity was assessed using real-time PCR. Libraries were sequenced on the Illumina Novaseq 6000 platform with paired-end runs of 150 bp read length. For each of the 32 samples, ~2.6 Gbp raw reads were generated (NCBI SRA: SRR18674070-101) (Supplementary Material S8, Table 1).

Data filtering, alignment, and genotype likelihood estimation

To account for the low coverage sequencing approach, we assessed genotype likelihoods using the Atlas Pipeline [106,107]. We first used the GAIA workflow to remove Illumina adapters with TrimGalore v0.6.6 (Trim Galore, RRID:SCR_011847)[108] with default parameters. Only reads longer than 30 bp were retained. Then, reads were aligned to the reference genome with BWA using BWA-MEM v0.7.17 (BWA, RRID:SCR_010910)[40] filtering for mapping quality scores >20. Alignments were processed with the RHEA workflow for indel realignment with GATK v3.8 (GATK, RRID:SCR_001876)[109]. A target interval set was created with a representative set of 15 samples and each individual was realigned together with a representative set of individuals (guidance samples) to enable realignment of low coverage samples without jointly realigning all samples. The average read depth per sample was 1.62–2.06 with a mean of 1.79 (Supplementary Material S8).

We used ANGSD v0.93 (ANGSD, RRID:SCR_021865)[110,111] to produce genotype likelihoods appropriate for the low coverage of individual samples. GATK was used (GATK, RRID:SCR_001876)[109] to infer major and minor alleles from the likelihoods (doMajorMinor 1, doMaf 1). Quality filtering for the subsequent downstream analyses was performed as follows: Only properly paired (only_proper_pairs 1) and unique reads (uniquieOnly 1) were used, and only biallelic sites were retained (skipTrialleleic 1). Nucleotides with base qualities below 20 were discarded. Excessive SNPs around indels and excessive mismatches with the reference were corrected (C50, baq 1,[112]. Sites with read coverage in fewer than 50% of the samples were excluded (minimum representation among samples > 50%, -minInd 16). SNPs with a genotype likelihood *p*-value < 0.001 were retained, producing a final set of 7,131,506 variant sites.

Population genetic structure and individual assignments

Our low coverage sequencing analyses focused on revealing within and among island genetic differentiation in the Aldabra Atoll, as well as assigning likely origins for zoo-housed individuals. We first assessed the global genetic structure of the samples using a principal component analysis with PCAngsd v09.85[113]. Based on a total of 6,651,907 variant sites with a minor allele frequency ≥0.05, individuals from Malabar and Grande Terre split into individual groups (Fig. 3B). Both zoo samples were grouped within the group of Grande Terre individuals revealing the most likely origin for these individuals captured in the 20th century. The principal component analysis also reveals a finer scale east-west population structure within islands confirming recent results based on ddRAD sequencing [114]**.**

We also assessed genetic structure using unsupervised Bayesian clustering with NGSAdmix (NGSadmix, RRID:SCR_003208)[115]. We performed pairwise linkage disequilibria (LD) pruning to reduce dependence among SNP loci[115]. Pairwise LD was calculated using ngsLD[116] and LD pruning was performed by allowing a maximum among-SNP distance of 100 Kbp and a minimum weight of 0.5. After LD pruning, 5,862,629 SNPs were retained and 50 replicate runs of NGSAdmix (NGSadmix, RRID:SCR_003208)[115] were performed. We varied the number of clusters (k) between 2-5 and visualized the assignments with PopHelper v.1.0.10[117,118] (Fig. 3C). The admixture analyses for *k*=2 clusters revealed a main split with groups formed by East Grande Terre together with East Malabar opposed to West Malabar. South & West Grande Terre individuals were assigned to both groups. At k=*4* each major sampling region was assigned to a single cluster. The zoo individual Maleika showed a genotype highly consistent with South & West Grande Terre individuals. The individual Hermania was assigned to different Grande Terre regions.

Variant annotation

Assessing the genetic health of a species is crucial to manage long-term survival. We analyzed mutation load by identifying putatively deleterious mutations segregating among Aldabra giant tortoises. We used SnpEff v5.1 (SnpEff, RRID:SCR_005191)[119,120] to functionally annotate all SNPs. SnpEff predicts the effects of genetic variants (e.g., loss of function). We identified SNPs using ANGSD v0.93 (ANGSD, RRID:SCR_021865)[110,111] as described above with the option -doBCF to create a BCF file. We converted the BCF to a VCF file with BCFtools v1.10.2 (SAMtools/BCFtools, RRID:SCR_005227)[121,122] applying a MAF filter of ≥0.05. The complete SNP dataset without a MAF filter yielded 7,131,506 SNPs where all SNPs had a minor allele frequency of ≥1%, whereas 6,651,907 SNPs were retained with MAF ≥0.05. We identified 1077 and 630 SNPs with a putatively high impact on gene function for the complete and MAF ≥0.05 datasets, respectively. For two SNP datasets, we identified 788 and 432 annotated as loss of function variants (*e.g.* mutated start or stop codons), and 325 and 124 were identified to have nonsense-mediated decays effect for the complete and MAF ≥0.05 datasets, respectively (Supplementary Material S9, S10). Analyzing whether selection is able to remove highly deleterious mutations will provide critical information on the ability of the species to retain high fitness over generations through purging.

Conclusions

We assembled the first high-quality, chromosome-level annotated genome for the Aldabra giant tortoise, resulting in one of the best assembled chelonian genomes. Chromosomal collinearity analyses revealed a high degree of conservation even among distantly related tortoise species. We showed that the high-quality resources can be combined with low-coverage resequencing to gain crucial insights into the genetic structure within the Aldabra

Atoll, as well as to resolve the exact origin of zoo-housed individuals. Understanding levels of genomic diversity in both native and ex-situ populations is crucial to inform rewilding efforts and prioritize conservation efforts. Furthermore, genome-wide analyses of polymorphism can be used to assess the presence of deleterious mutations endangering the long-term health of populations. Finally, given the exceptionally long lifespan and large body size, the high-quality genome will inform comparative genomics studies focused on the genetic underpinnings of aging and gigantism.

Data availability

The raw sequencing data, the nuclear and mitochondrial genome assemblies, and the annotation produced in this study have been deposited in the NCBI under BioProject accession number PRJNA822095. They will be available upon the acceptance of the manuscript.

Additional Files

Supplementary Material S1. K-mer (k=17) profile of the *Aldabrachelys gigantea* genome. Consistent with low heterozygosity, most of the k-mers form one peak centered around roughly 20× coverage and do not form another peak centered at roughly half the coverage that would represent k-mers arising from heterozygous alleles.

Supplementary Material S2. Genome contiguity statistics of the assemblies obtained from different assemblers. The column shaded in gray represents our initial assembly obtained via default parameters in Hifiasm

Supplementary Material S3. Summary of repeat annotations

Supplementary Material S4. Accession details of the short read RNA-seq samples used in this study

Supplementary Material S5. BUSCO statistics for the protein coding gene annotation of *Aldabrachelys gigantea*, *Chelonoidis abingdonii*, and *Gopherus evgoodei*

Supplementary Material S6. Summary statistics of the functionally annotated protein-coding genes

Supplementary Material S7. Circos plot showing the synteny between the *Aldabrachelys gigantea* Hi-C scaffolds (orange) and *Gopherus evgoodei* assembly pseudo-chromosomes (green).

Supplementary Material S8. Details of the location of 30 low coverage whole genome resequencing samples

Supplementary Material S9. Numbers of annotated SNPs with no MAF filtering and MAF ≥0.05 by their impact

Supplementary Material S10. Percentage of effects by their region on the genome A) effects of SNPs with no MAF filter B) MAF≥0.05

Abbreviations

µg, microgram; µl, microliter; °C, degree Celcius; AGAT, Another Gtf/Gff Analysis Toolkit; ANGSD, Analysis of Next Generation Sequencing Data; baq, base alignment quality; bp, base pairs; BUSCO, Benchmarking Universal Single-Copy Orthologs; BWA, Burrows-Wheeler Aligner; DNA, deoxyribonucleic acid; cDNA, complementary DNA;

dsDNA, double-strand DNA; EAZA, European Association of Zoos and Aquaria; ETH, Swiss Federal Institute of Technology in Zürich; GAIA, Genome-wide Alignment Including Adapter-trimming; GATK, Genome Analysis Toolkit; Gbp, gigabase pairs; GC, guanine and cytosine; GCE, Genomic Character Estimator; gDNA, genomic DNA; Hi-C, chromosome conformation capture; HiFi, high-fidelity; HMW, high molecular weight; IsoSeq, isoform sequencing; IUCN, International Union for Conservation of Nature; JBAT, Juicebox Assembly Tools; KAT, k-mer

analysis toolkit; LS, liquid sample; MAF, minor allele frequency; Mbp, megabase pairs; mg, milligram; min, minute; mL, milliliter; mM, millimolar; NCBI, National Center for Biotechnology Information; NEB, New England Biolabs; ng, nanogram; NGSAdmix, Next Generation Sequencing Admixture; ngsLD, Next Generation Sequencing Linkage Disequilibrium; OrthoDB, orthologous database; PacBio, Pacific Biosciences; PBS, phosphate-buffered saline; PCA, principal component analysis; PCR, polymerase chain reaction; QUAST, Quality Assessment Tool; RefSeq, reference sequence; Rfam, RNA families; RNA, ribonucleic acid; RNA-seq, RNA sequencinf; rpm, revolutions per minute; Sauropsida_odb10, sauropsids orthologous database 10; SMRT, single-molecule real-time; SNP, single nucleotide polymorphism; SRA, Sequence Read Archive; STAR, Spliced Transcripts Alignment to a Reference; SyRI, Synteny and Rearrangement Identifier; tRNA, transfer RNA; rRNA, ribosomal RNA; snRNA, small nuclear RNA; miRNA, micro RNA; TSEBRA, Transcript Selector for BRAKER; UniProtKB, Universal Protein Knowledgebase; Vertebrata_odb10, vertebrate orthologous database 10

Competing interests

The authors declare that they have no competing interests.

Funding

This study was funded through the Research Talent Development Fund of the University of Zürich, the Swiss National Science Foundation (Project No: 31003A_182343), and University of Zurich Internal Funds, all of which were given to C.G.

Author contributions

F.G.Ç. and C.G conceived the study design. F.G.Ç. carried out all DNA and RNA extractions and bioinformatic analyses with guidance from D.C. and C.G. D.H. and L.D. coordinated the sampling of the zoo animals. N.B. helped get permits required for tortoise sampling on Aldabra Atoll and L.A. managed the collection, storage, and transport of samples from wild individuals. F.G.Ç. wrote the manuscript with guidance from C.G and substantial input from D.C. All authors revised the manuscript.

Acknowledgments

We gratefully acknowledge Jean-Michel Hatt, Gabriela Hurlimann, and Maya Kummrow for facilitating sample donation, Claudia Rudolf von Rohr, and the team of tortoise keepers at the Masoala Rainforest in Zurich Zoo for their assistance and helpful discussions. We thank the Seychelles Islands Foundation staff Maria Bielsa, Mickael Esparon, Bruno Mels, Martin van Rooyen, Mersiah Rose, and Brian Souyana for sample collection on Aldabra Atoll; Ronny Rose and Frauke Fleischer-Dogley for their assistance with the handling, storage, and transport of the samples. We also thank Sirpa Kurz from the Zoological Museum, University of Zurich, and Constantin Latt from the Natural History Museum of Bern for their help in tissue sampling of the tortoise Maleika. Additionally, we thank Silvia Kobel and Aria Minder from the Genetic Diversity Centre, ETH Zurich for their help in wet lab applications. We also thank Simon Grüter and Weihong Qi from the Functional Genomics Center, ETH, Zurich for their help in getting sequencing services.

References

1. Barnosky AD, Matzke N, Tomiya S, Wogan GOU, Swartz B, Quental TB, Ferrer, E. A. Has the Earth's sixth mass extinction already arrived? Nature. 2011; 471(7336):51-57.

2. Formenti G, Theissinger K, Fernandes C, Bista I, Bombarely A, Bleidorn C, et al. The era of reference genomes in conservation genomics. Trends Ecol Evol. 2022; 37:197–202.
3. Harris A. Lewin, Gene E. Robinson, W. John Kress, William J. Baker, Jonathan Coddington, Keith A. Crandall, Richard Durbin, Scott V. Edwards, Félix Forest, M. Thomas P. Gilbert, Melissa M. Goldstein, Igor V. Grigoriev, Kevin J. Hackett, David Haussler, Erich D. Jarvis, Warren E. Johnson, Aristides Patrinos, Stephen Richards, Juan Carlos Castilla-Rubio, Marie-Anne van Sluys, Pamela S. Soltis, Xun Xu, Huanming Yang, and Guojie Zhang Earth BioGenome Project: Sequencing life for the future of life. PNAS. 2018; 115:4325–33.
4. Genome 10K Community of Scientists. Genome 10K: A Proposal to Obtain Whole-Genome Sequence for 10 000 Vertebrate Species. J Hered. 2009; 100:659–74.
5. Koepfli K-P, Paten B, Genome 10K Community of Scientists, O'Brien SJ. The Genome 10K Project: a way forward. Annu Rev Anim Biosci. 2015; 3:57–111.
6. GIGA Community of Scientists. The Global Invertebrate Genomics Alliance (GIGA): Developing Community Resources to Study Diverse Invertebrate Genomes. J Hered. 2013; 105:1–18.
7. Shaffer HB, McCartney-Melstad E, Near TJ, Mount GG, Spinks PQ. Phylogenomic analyses of 539 highly informative loci dates a fully resolved time tree for the major clades of living turtles (Testudines). Mol Phylogenet Evol. 2017; 115:7–15.
8. Lovich JE, Ennen JR, Agha M, Gibbons JW. Where have all the turtles gone, and why does it matter? Bioscience. 2018; 68:771–81.
9. Gibbons JW. Why Do Turtles Live So Long? Bioscience. 1987; 37:262–9.
10. Turtle Taxonomy Working Group [Rhodin, A.G.J., Iverson, J.B., Bour, R., Fritz, U., Georges, A., Shaffer, H.B., and van Dijk, P.P.]. 2021. Turtles of the World: Annotated Checklist and Atlas of Taxonomy, Synonymy, Distribution, and Conservation Status (9th Ed.). In: Rhodin, A.G.J., Iverson, J.B., van Dijk, P.P., Stanford, C.B., Goode, E.V.,

Buhlmann, K.A., and Mittermeier, R.A. (Eds.). Conservation Biology of Freshwater Turtles and Tortoises: A Compilation Project of the IUCN/SSC Tortoise and Freshwater Turtle Specialist Group. Chelonian Research Monographs. 2021; 8:1–472.

11. van Oosterhout C. Mutation load is the spectre of species conservation. Nat Ecol Evol. 2020; 4:1004–6.

12. Quesada V, Freitas-Rodríguez S, Miller J, Pérez-Silva JG, Jiang Z-F, Tapia W, et al.. Giant tortoise genomes provide insights into longevity and age-related disease. Nat Ecol Evol. 2019; 3:87–95.

13. Hansen DM, Josh Donlan C, Griffiths CJ, Campbell KJ. Ecological history and latent conservation potential: large and giant tortoises as a model for taxon substitutions. Ecography. 2010; 33:272–84.

14. Hnatiuk RJ, Woodell SRJ, Bourn DM. Giant tortoise and vegetation interactions on Aldabra Atoll—Part 2: coastal. Biol Conserv. 1976; 9:305–16.

15. Merton LFH, Bourn DM, Hnatiuk RJ. Giant tortoise and vegetation interactions on Aldabra Atoll—Part 1: inland. Biol Conserv. 1976; 9:293–304.

16. Hansen DM. Non-native megaherbivores: the case for novel function to manage plant invasions on islands. AoB Plants. 2015; 7:lv085.

17. Falcón W, Moll D, Hansen DM. Frugivory and seed dispersal by chelonians: a review and synthesis. Biol Rev Camb Philos Soc. 2020; 95:142–66.

18. Griffiths O, Andre A, Meunier A. Tortoise breeding and “re-wilding” on Rodrigues Island. Chelonian Res Monogr. 2013; 6:178–82.

19. Pedrono M, Griffiths OL, Clausen A, Smith LL, Griffiths CJ, Wilmé L, et al.. Using a surviving lineage of Madagascar’s vanished megafauna for ecological restoration. Biol Conserv. 2013; 159:501–6.

20. Frankham R, Ballou SEJ, Briscoe DA, Ballou JD. Introduction to Conservation Genetics. Cambridge University Press. 2002.
21. Austin JJ, Nicholas Arnold E, Bour R. Was there a second adaptive radiation of giant tortoises in the Indian Ocean? Using mitochondrial DNA to investigate speciation and biogeography of Aldabrachelys (Reptilia, Testudinidae). Mol Ecol. 2003; 12:1415–24.
22. Palkovacs EP, Marschner M, Ciofi C, Gerlach J, Caccone A. Are the native giant tortoises from the Seychelles really extinct? A genetic perspective based on mtDNA and microsatellite data. Mol Ecol. 2003; 12:1403–13.
23. Sim SB, Corpuz RL, Simmonds TJ, Geib SM. HiFiAdapterFilt, a memory efficient read processing pipeline, prevents occurrence of adapter sequence in PacBio HiFi reads and their negative impacts on genome assembly. BMC Genomics. 2022; 23:157.
24. Sim SB. HiFiAdapterFilt (Version 2.0.0) https://github.com/sheinasim/HiFiAdapterFilt/releases/tag/v2.0.0
25. Liu B, Shi Y, Yuan J, Hu X, Zhang H, Li N, et al. Estimation of genomic characteristics by analyzing k-mer frequency in de novo genome projects. arXiv. 2013; https://doi.org/10.48550/arXiv.1308.2012.
26. Liu B, Shi Y, Yuan J, Hu X, Zhang H, Li N, et al. (2013) GCE (Version 1.0.2) https://github.com/fanagislab/GCE
27. Nurk S, Walenz BP, Rhie A, Vollger MR, Logsdon GA, Grothe R, et al. HiCanu: accurate assembly of segmental duplications, satellites, and allelic variants from high-fidelity long reads. Genome Res. 2020; 30:1291–305.
28. Nurk S, Walenz BP, Rhie A, Vollger MR, Logsdon GA, Grothe R, et al. (2020). HiCanu (Version 2.2) https://github.com/marbl/canu/releases/tag/v2.2
29. Sovic I, Kronenberg Z, Dunn C, Barnett D, Kingan S, Drake J (2020). IPA HiFi Genome Assembler (Version 1.8.0) https://github.com/PacificBiosciences/pbipa/releases/tag/v1.8.0

30. Cheng H, Concepcion GT, Feng X, Zhang H, Li H. Haplotype-resolved de novo assembly using phased assembly graphs with hifiasm. Nat Methods. 2021; 18:170–5.
31. Cheng H, Concepcion GT, Feng X, Zhang H, Li H (2021). Hifiasm (Version 0.15) https://github.com/chhylp123/hifiasm/releases/tag/0.15.5
32. Mikheenko A, Prjibelski A, Saveliev V, Antipov D, Gurevich A. Versatile genome assembly evaluation with QUAST-LG. Bioinformatics. 2018; 34(13):i142–50.
33. Mikheenko A, Prjibelski A, Saveliev V, Antipov D, Gurevich A. (2018). QUAST (Version 5.0.2) https://github.com/ablab/quast/releases/tag/quast_5.0.2
34. Shen W, Le S, Li Y, Hu F. SeqKit: A Cross-Platform and Ultrafast Toolkit for FASTA/Q File Manipulation. PLoS One. 2016; 11:e0163962.
35. Shen W, Le S, Li Y, Hu F. (2021) SeqKit (Version 0.16.1) https://github.com/shenwei356/seqkit/releases/tag/v0.16.1
36. Buchfink B, Reuter K, Drost H-G. Sensitive protein alignments at tree-of-life scale using DIAMOND. Nat Methods. 2021; 18:366–8.
37. Buchfink B, Reuter K, Drost H-G (2021) diamond (Version 2.0.9) https://github.com/bbuchfink/diamond/releases/tag/v2.0.9
38. Mapleson D, Garcia Accinelli G, Kettleborough G, Wright J, Clavijo BJ. KAT: a K-mer analysis toolkit to quality control NGS datasets and genome assemblies. Bioinformatics. 2017; 33:574–6.
39. Mapleson D, Garcia Accinelli G, Kettleborough G, Wright J, Clavijo BJ. (2018). KAT (Version 2.4.1) https://github.com/TGAC/KAT/releases/tag/Release-2.4.1
40. Li H, Durbin R. Fast and accurate short read alignment with Burrows-Wheeler transform. Bioinformatics. 2009; 25:1754–60.

41. Okonechnikov K, Conesa A, García-Alcalde F. Qualimap 2: advanced multi-sample quality control for high-throughput sequencing data. Bioinformatics. 2016; 32:292–4.
42. Okonechnikov K, Conesa A, García-Alcalde F. (2016). Qualimap (Version 2.2.1) http://qualimap.conesalab.org/archive.html
43. Seppey M, Manni M, Zdobnov EM. BUSCO: Assessing Genome Assembly and Annotation Completeness. Methods Mol Biol. 2019; 1962:227–45.
44. Seppey M, Manni M, Zdobnov EM. (2021) BUSCO (Version 5.1.2) https://gitlab.com/ezlab/busco/-/releases/5.1.2
45. Durand NC, Shamim MS, Machol I, Rao SSP, Huntley MH, Lander ES, et al. Juicer Provides a One-Click System for Analyzing Loop-Resolution Hi-C Experiments. Cell Syst. 2016; 3:95–8.
46. Durand NC, Shamim MS, Machol I, Rao SSP, Huntley MH, Lander ES, et al. (2020) Juicer (Version 1.6) https://github.com/aidenlab/juicer/releases/tag/1.6
47. Dudchenko O, Batra SS, Omer AD, Nyquist SK, Hoeger M, Durand NC, et al. De novo assembly of the Aedes aegypti genome using Hi-C yields chromosome-length scaffolds. Science. 2017; 356:92–5.
48. Dudchenko O, Batra SS, Omer AD, Nyquist SK, Hoeger M, Durand NC, et al. (2021) 3D DNA (Phasing branch 201008) https://github.com/aidenlab/3d-dna/releases/tag/201008

49. Durand NC, Robinson JT, Shamim MS, Machol I, Mesirov JP, Lander ES, et al. Juicebox Provides a Visualization System for Hi-C Contact Maps with Unlimited Zoom. Cell Syst. 2016; 3:99–101.
50. Challis R, Richards E, Rajan J, Cochrane G, Blaxter M. BlobToolKit--interactive quality assessment of genome assemblies. G3: Genes, Genomes, Genetics. 2020; 10:1361–74.

51. Challis R, Richards E, Rajan J, Cochrane G, Blaxter M. (2021). BlobToolKit (Version 2.6.4) https://github.com/blobtoolkit/pipeline/releases/tag/2.6.4

52. Flynn JM, Hubley R, Goubert C, Rosen J, Clark AG, Feschotte C, et al. RepeatModeler2 for automated genomic discovery of transposable element families. PNAS. 2020; 117:9451–7.

53. Hubley R, Smit A (2021). RepeatModeler (Version 2.02a) https://www.repeatmasker.org/RepeatModeler/RepeatModeler-2.0.2a.tar.gz

54. Bao Z, Eddy SR. Automated de novo identification of repeat sequence families in sequenced genomes. Genome Res. 2002; 12:1269–76.

55. Price AL, Jones NC, Pevzner PA. De novo identification of repeat families in large genomes. Bioinformatics. 2005; 21:351–8.

56. Benson G. Tandem repeats finder: a program to analyze DNA sequences. Nucleic Acids Res. 1999; 27:573–80.

57. RepeatMasker (2020). RepeatMasker (Version 4.1.0). http://www.repeatmasker.org/RepeatMasker/RepeatMasker-4.1.0.tar.gz

58. Brůna T, Hoff KJ, Lomsadze A, Stanke M, Borodovsky M. BRAKER2: automatic eukaryotic genome annotation with GeneMark-EP+ and AUGUSTUS supported by a protein database. NAR Genom Bioinform. 2021; 3:lqaa108.

59. Hoff KJ, Lomsadze A, Borodovsky M, Stanke M. Whole-Genome Annotation with BRAKER. Methods Mol Biol. 2019; 1962:65–95.

60. Hoff KJ, Lange S, Lomsadze A, Borodovsky M, Stanke M. BRAKER1: Unsupervised RNA-Seq-Based Genome Annotation with GeneMark-ET and AUGUSTUS. Bioinformatics. 2016; 32:767–9.

61. Stanke M, Diekhans M, Baertsch R, Haussler D. Using native and syntenically mapped cDNA alignments to improve de novo gene finding. Bioinformatics. 2008; 24:637–44.

62. Brůna T, Hoff KJ, Lomsadze A, Stanke M, Borodovsky M (2020). BRAKER2 (Version 2.1.5) https://github.com/Gaius-Augustus/BRAKER/releases/tag/v2.1.5

63. Dobin A, Davis CA, Schlesinger F, Drenkow J, Zaleski C, Jha S, et al. STAR: ultrafast universal RNA-seq aligner. Bioinformatics. 2013; 29:15–21.

64. Dobin A, Davis CA, Schlesinger F, Drenkow J, Zaleski C, Jha S, et al. (2021) STAR (Version 2.7.9) https://github.com/alexdobin/STAR/releases/tag/2.7.9a

65. Li H. Minimap2: pairwise alignment for nucleotide sequences. Bioinformatics. 2018; 34:3094–100.

66. Li H (2021) minimap2 (Version 2.24) https://github.com/lh3/minimap2/releases/tag/v2.24

67. Kriventseva EV, Kuznetsov D, Tegenfeldt F, Manni M, Dias R, Simão FA, et al. OrthoDB v10: sampling the diversity of animal, plant, fungal, protist, bacterial and viral genomes for evolutionary and functional annotations of orthologs. Nucleic Acids Res. 2019; 47:D807–11.

68. Brůna T, Lomsadze A, Borodovsky M. GeneMark-EP+: eukaryotic gene prediction with self-training in the space of genes and proteins. NAR Genom Bioinform. 2020; 2:lqaa026.

69. Brůna T, Lomsadze A, Borodovsky M (2021) ProtHint (Version 2.6.0) https://github.com/gatech-genemark/ProtHint/releases/tag/v2.6.0

70. Buchfink B, Xie C, Huson DH. Fast and sensitive protein alignment using DIAMOND. Nat Methods. 2015; 12:59–60.

71. Lomsadze A, Ter-Hovhannisyan V, Chernoff YO, Borodovsky M. Gene identification in novel eukaryotic genomes by self-training algorithm. Nucleic Acids Res. 2005; 33:6494–506.

72. Iwata H, Gotoh O. Benchmarking spliced alignment programs including Spaln2, an extended version of Spaln that incorporates additional species-specific features. Nucleic Acids Res. 2012; 40:e161.

73. Gotoh O, Morita M, Nelson DR. Assessment and refinement of eukaryotic gene structure prediction with gene-structure-aware multiple protein sequence alignment. BMC Bioinformatics. 2014; 15:189.
74. Lomsadze A, Burns PD, Borodovsky M. Integration of mapped RNA-Seq reads into automatic training of eukaryotic gene finding algorithm. Nucleic Acids Res. 2014; 42:e119.
75. Gabriel L, Hoff KJ, Brůna T, Borodovsky M, Stanke M. TSEBRA: transcript selector for BRAKER. BMC Bioinformatics. 2021; 22(1):566.
76. Gabriel L, Hoff KJ, Brůna T, Borodovsky M, Stanke M. (2021) TSEBRA (Version 1.0.3) https://github.com/Gaius-Augustus/TSEBRA/releases/tag/v1.0.3
77. Haas B (2010) TransposonPSI: An Application of PSI-Blast to Mine (Retro-)Transposon ORF Homologies. http://transposonpsi.sourceforge.net/ Accessed 10 August 2021
78. Jones P, Binns D, Chang H-Y, Fraser M, Li W, McAnulla C, et al. InterProScan 5: genome-scale protein function classification. Bioinformatics. 2014; 30:1236–40.
79. Jones P, Binns D, Chang H-Y, Fraser M, Li W, McAnulla C, et al. (2021) Interproscan (Version 5.53-87.0) https://github.com/ebi-pf-team/interproscan/releases/tag/5.53-87.0
80. Boutet E, Lieberherr D, Tognolli M, Schneider M, Bansal P, Bridge AJ, et al. UniProtKB/Swiss-Prot, the Manually Annotated Section of the UniProt KnowledgeBase: How to Use the Entry View. Methods Mol Biol. 2016; 1374:23–54.
81. Dainat J (2021, Nov 22) AGAT: Another Gff Analysis Toolkit to Handle Annotations in Any GTF/GFF Format (Version v0. 5.1). Zenodo. https://www.doi.org/10.5281/zenodo.3552717.
82. Dainat J (2021) AGAT (Version 0.8.0) https://github.com/NBISweden/AGAT/releases/tag/v0.8.0
83. Nawrocki EP, Eddy SR. Infernal 1.1: 100-fold faster RNA homology searches. Bioinformatics. 2013; 29:2933–5.

84. Nawrocki EP, Eddy SR. (2020) Infernal (Version 1.1.4) https://github.com/EddyRivasLab/infernal/releases/tag/infernal-1.1.4

85. Nawrocki EP, Burge SW, Bateman A, Daub J, Eberhardt RY, Eddy SR, et al. Rfam 12.0: updates to the RNA families database. Nucleic Acids Res. 2015; 43:D130–7.

86. Allio R, Schomaker-Bastos A, Romiguier J, Prosdocimi F, Nabholz B, Delsuc F. MitoFinder: Efficient automated large-scale extraction of mitogenomic data in target enrichment phylogenomics. Mol Ecol Resour. 2020; 20:892–905.

87. Allio R, Schomaker-Bastos A, Romiguier J, Prosdocimi F, Nabholz B, Delsuc F. (2021) MitoHiFi (Version 2.0) https://github.com/marcelauliano/MitoHiFi/releases/tag/v2.0

88. Besnard G, Thèves C, Mata X, Holota H, Rakotozafy LMA, Pedrono M. Shotgun sequencing of the mitochondrial genome of the Aldabra giant tortoise (Aldabrachelys gigantea). Mitochondrial DNA. 2016; 27:4543–4.

89. Goel M, Sun H, Jiao W-B, Schneeberger K. SyRI: finding genomic rearrangements and local sequence differences from whole-genome assemblies. Genome Biol. 2019; 20:277.

90. Goel M, Sun H, Jiao W-B, Schneeberger K (2022). SyRI (Version 1.5.4) https://github.com/schneebergerlab/syri/releases/tag/v1.5.421.

91. Goel M, Schneeberger K. plotsr: Visualising structural similarities and rearrangements between multiple genomes. bioRxiv. 2022; doi: 10.1101/2022.01.24.477489.

92. Goel M, Schneeberger K (2022). plotsr (Version 0.5.3) https://github.com/schneebergerlab/plotsr/releases/tag/v0.5.3

93. Kehlmaier C, Graciá E, Campbell PD, Hofmeyr MD, Schweiger S, Martínez-Silvestre A, et al. Ancient mitogenomics clarifies radiation of extinct Mascarene giant tortoises (Cylindraspis spp.). Sci Rep. 2019; 9:17487.

94. Ren Y, Zhang Q, Yan X, Hou D, Huang H, Li C, et al. Genomic insights into the evolution of the critically endangered soft-shelled turtle Rafetus swinhoei. Mol Ecol Resour. 2022; doi: 10.1111/1755-0998.13596.

95. Emms DM, Kelly S. OrthoFinder: phylogenetic orthology inference for comparative genomics. Genome Biol. 2019; 20:238.

96. Emms DM, Kelly S (2021). OrthoFinder (Version 2.5.4) https://github.com/davidemms/OrthoFinder/releases/tag/2.5.4

97. Vandepoele K, Saeys Y, Simillion C, Raes J, Van De Peer Y. The automatic detection of homologous regions (ADHoRe) and its application to microcolinearity between Arabidopsis and rice. Genome Res. 2002; 12:1792–801.

98. Vandepoele K, Saeys Y, Simillion C, Raes J, Van De Peer Y. (2020). i-ADHoRe (Version 3.0) https://github.com/VIB-PSB/i-ADHoRe/releases/tag/3.0

99. Krzywinski M, Schein J, Birol I, Connors J, Gascoyne R, Horsman D, et al. Circos: an information aesthetic for comparative genomics. Genome Res. 2009; 19:1639–45.

100. Krzywinski M, Schein J, Birol I, Connors J, Gascoyne R, Horsman D, et al. (2019) Circos (Version 0.69-8). http://circos.ca/distribution/circos-0.69-8.tgz

101. Bourn D, Coe M. The size, structure and distribution of the giant tortoise population of Aldabra. Philos Trans R Soc Lond B Biol Sci. 1978; 282:139–75.

102. Bourn D, Gibson C, Augeri D, Wilson CJ, Church J, Hay SI. The rise and fall of the Aldabran giant tortoise population. Proceedings of the Royal Society B: Biological Sciences. 1999; 266:1091–100.

103. Lou RN, Jacobs A, Wilder A, Therkildsen NO. A beginner’s guide to low-coverage whole genome sequencing for population genomics. Molecular Ecology Resources. 2021; 30:5966–93.

104. Orlando L, Allaby R, Skoglund P, Der Sarkissian C, Stockhammer PW, Ávila-Arcos MC, et al. Ancient DNA analysis. Nature Reviews Methods Primers. 2021; 1:1–26.
105. Species360 Zoological Information Management System (ZIMS) (2017). zims.Species360.org Accessed 1 Apr 2022.
106. Wegmann D. ATLAS-Pipeline: a flexible aDNA-pipeline using Atlas and Snakemake https://bitbucket.org/wegmannlab/atlas-pipeline/src/master/ Accessed 2 September 2021.
107. Link V, Kousathanas A, Veeramah K, Sell C, Scheu A, Wegmann D. ATLAS: Analysis tools for low-depth and ancient samples. bioRxiv. 2017; https://doi.org/10.1101/105346.
108. Krueger F (2020) TrimGalore (Version 0.6.6) https://github.com/FelixKrueger/TrimGalore/releases/tag/0.6.6
109. McKenna A, Hanna M, Banks E, Sivachenko A, Cibulskis K, Kernytsky A, et al. The Genome Analysis Toolkit: a MapReduce framework for analyzing next-generation DNA sequencing data. Genome Res. 2010; 20:1297–303.
110. Korneliussen TS, Albrechtsen A, Nielsen R. ANGSD: analysis of next generation sequencing data. BMC Bioinformatics. 2014; 15:356.
111. Korneliussen TS, Albrechtsen A, Nielsen R. (2019) ANGSD (Version 0.93) https://github.com/ANGSD/angsd/releases/tag/0.930
112. Li H. Improving SNP discovery by base alignment quality. Bioinformatics. 2011; 27:1157–8.
113. Meisner J, Albrechtsen A. Inferring population structure and admixture proportions in low-depth NGS data. Genetics. 2018; 210:719–31.
114. Çilingir FG, Hansen D, Bunbury N, Postma E, Baxter R, Turnbull L, et al. Low-coverage reduced representation sequencing reveals subtle within-island genetic structure in Aldabra giant tortoises. Ecol Evol. 2022; 12:e8739.

115. Skotte L, Korneliussen TS, Albrechtsen A. Estimating individual admixture proportions from next generation sequencing data. Genetics. 2013; 195:693–702.
116. Fox EA, Wright AE, Fumagalli M, Vieira FG. ngsLD: evaluating linkage disequilibrium using genotype likelihoods. Bioinformatics. 2019; 35:3855–6.
117. Francis RM. pophelper: an R package and web app to analyse and visualize population structure. Mol Ecol Resour. 2017; 17:27–32.
118. Francis R: Pophelper. http://www.royfrancis.com/pophelper/articles/ Accessed 2021 Oct 23.
119. Cingolani P, Platts A, Wang LL, Coon M, Nguyen T, Wang L, et al. A program for annotating and predicting the effects of single nucleotide polymorphisms, SnpEff: SNPs in the genome of Drosophila melanogaster strain w1118. Fly (Austin). 2012; 6:80–92
120. Cingolani P, Platts A, le L W, Coon M, Nguyen T, Wang L, et al. (2017) SnpEff (Version 5.1)
https://sourceforge.net/projects/snpeff/files/
121. Danecek P, Bonfield JK, Liddle J, Marshall J, Ohan V, Pollard MO, et al. Twelve years of SAMtools and BCFtools. Gigascience. 2021; https://doi.org/10.1093/gigascience/giab008.
122. Danecek P, Bonfield JK, et al. (2019) BCFtools (Version 1.10.2).
https://github.com/samtools/bcftools/releases/tag/1.10.2

Figure Legends

Fig. 1 A) A female *Aldabrachelys gigantea* resting at La Vanille Nature Park, Mauritius. **B)** World map showing the location of Aldabra Atoll. **C)** Hi-C contact map of the chromosome-level assembled *Aldabrachelys gigantea* reference genome. Blue boxes represent assembled pseudo-chromosomes and green boxes represent assembled scaffolds that constitute pseudo-chromosomes. **D)** BUSCO completeness scores for the sauropsida dataset and **E)** Assembly

metrics (including length of the longest scaffold N50 and N90) and sequence composition (GC content) of the chromosome-level *Aldabrachelys gigantea* genome.

Fig. 2 Synteny analysis of 10 chromosomes in *Aldabrachelys gigantea* (blue horizontal lines), *Gopherus evgoodei* (orange horizontal lines), *Mauremys mutica* (green horizontal lines), and *Trachemys scripta elegans* (red horizontal lines) genome assemblies shows high levels of conservation between distantly related chelonian taxa. Gray, yellow, green, and light blue lines between assemblies indicate syntenic regions, inversions, translocations, and duplications, respectively. The four compared assemblies represent all chelonian families (see cladogram on the left, split times from [93]) except Platysternidae within the chelonian super family Testudinoidea, which includes families of Emydidae (terrapins), Geoemydidae and Testudinidae (land-dwelling tortoises). *Trachemys scripta elegans* is from Emydidae, *Mauremys mutica* is from Geoemydidae, and *Gopherus evgoodei* and *Aldabrachelys gigantea* are from Testudinidae.

Fig. 3 A) The location of the Aldabra Atoll in the Western Indian Ocean and sampling location of 30 individuals within Aldabra Atoll. Every colored mark on the map represents a sampled tortoise. **B)** Principal component analysis plot of 30 *Aldabrachelys gigantea* individuals from the islands of Grande Terre (East, light blue circles; South & West, green circles) and Malabar (East, dark blue triangles; West, purple triangles) and two individuals from the Zurich Zoo (Hermania, black diamond; Maleika, light pink square). Principal components 1 and 2 account for 14.2% and 3.64% of the overall genetic variability, respectively. **C)** Admixture proportions of all the individuals for ancestral populations (k) varied from 2 to 5. Each bar represents one individual and shows its admixture proportions.

 Click here to access/download;Table;Table_1.docx

Table 1 Summary of the genomic data produced in this study

Aldabrachelys gigantea **reference genome sequencing, assembly, and validation**	
NCBI BioProject	PRJNA822095
**Draft genome sequencing**	
PacBio SMRT II HiFi data (Gb)	192
HiFi reads NCBI SRA Accession	SRR18672579
**Hi-C scaffolding**	
Illumina NovaSeq 6000 data (Gb)	196
Hi-C reads SRA Accession	SRR18673000
**Chromosome-level genome assembly (AldGig_1.0)**	
Assembled genome size (Gb)	2.37
Scaffold N50 (Mb)	148.6
No. of scaffolds	719
Contig N50 (Mb)	61.5
No. of contigs	422
BUSCO completeness (sauropsida_odb10)	97.3% complete, 0.4% fragmented, 2.3% missing
**Mitochondrial genome assembly**	
Assembled genome size (bp)	16,467
**Genome annotation**	
PacBio SMRT IsoSeq data (Gb)	1.1
IsoSeq reads NCBI SRA Accession	SRR18674283
No. of predicted protein-coding genes	23,953
No. of functionally annotated genes	22,554
BUSCO completeness (sauropsida_odb10)	91.9% complete, 2.3% fragmented, 5.8% missing
DOI for annotations	doi.org/10.5281/zenodo.6528994
**Low coverage whole-genome resequencing**	
Illumina NovaSeq 6000 data (Gb)	202
NCBI SRA Accessions	SRR14611971-SRR18674101

 Click here to access/download;Table;Table_2.docx

Table 2 Contiguity and completeness statistics of all available chromosome-level assembled chelonian genomes

Species name (Accession No)	Family	Genome size (Gbp)	Contig N50 (Mbp)	Scaffold N50 (Mbp)	BUSCO completeness*
Aldabrachelys gigantea (This study)	Testudinidae	2.374	61.5	148.6	97.3%[S:96.2%,D:1.1%],F:0.4%,M:2.3%
Gopherus evgoodei (GCF_007399415.2)	Testudinidae	2.299	13.027	147.4	97%[S:95.9,D:1.1%],F:0.5%,M:2.5%
Chelonia mydas (GCF_015237465.2)	Cheloniidae	2.134	39.416	134.4	97.3%[S:96.3%,D:1.0%],F:0.4%,M:2.3%
Dermochelys coriacea (GCF_009764565.3)	Dermochelyidae	2.165	7.03	137.6	96.3%[S:95.3%,D:1.0%],F:0.6%,M:3.1%
Chrysemys picta bellii (GCF_000241765.4)	Emydidae	2.481	0.021	16	96.6%[S:95.7%,D:0.9%],F:1.1%,M:2.3%
Trachemys scripta elegans (GCF_013100865.1)	Emydidae	2.126	0.205	140.4	95.0%[S:94.0%,D:1.0%],F:1.2%,M:3.8%
Mauremys mutica (GCF_020497125.1)	Geoemydidae	2.484	15.011	135	97.3%[S:95.2%,D:2.1%],F:0.5%,M:2.2%
Mauremys reevesii (GCF_016161935.1)	Geoemydidae	2.368	33.353	130.5	97.5%[S:95.9%,D:1.6%],F:0.4%,M:2.1%
Rafetus swinhoei (GCA_019425775.1)	Trionychidae	2.238	30.964	132	96.4%[S:95.3%,D:1.1%],F:0.6%,M:3.0%

*BUSCO score generated from the sauropsid (sauropsida_odb10) database. BUSCO statistics C, complete; S, single-copy; D, duplicated; F, fragmented; M, missing

Figure 1

Click here to access/download;Figure;Figure1_sm.pdf

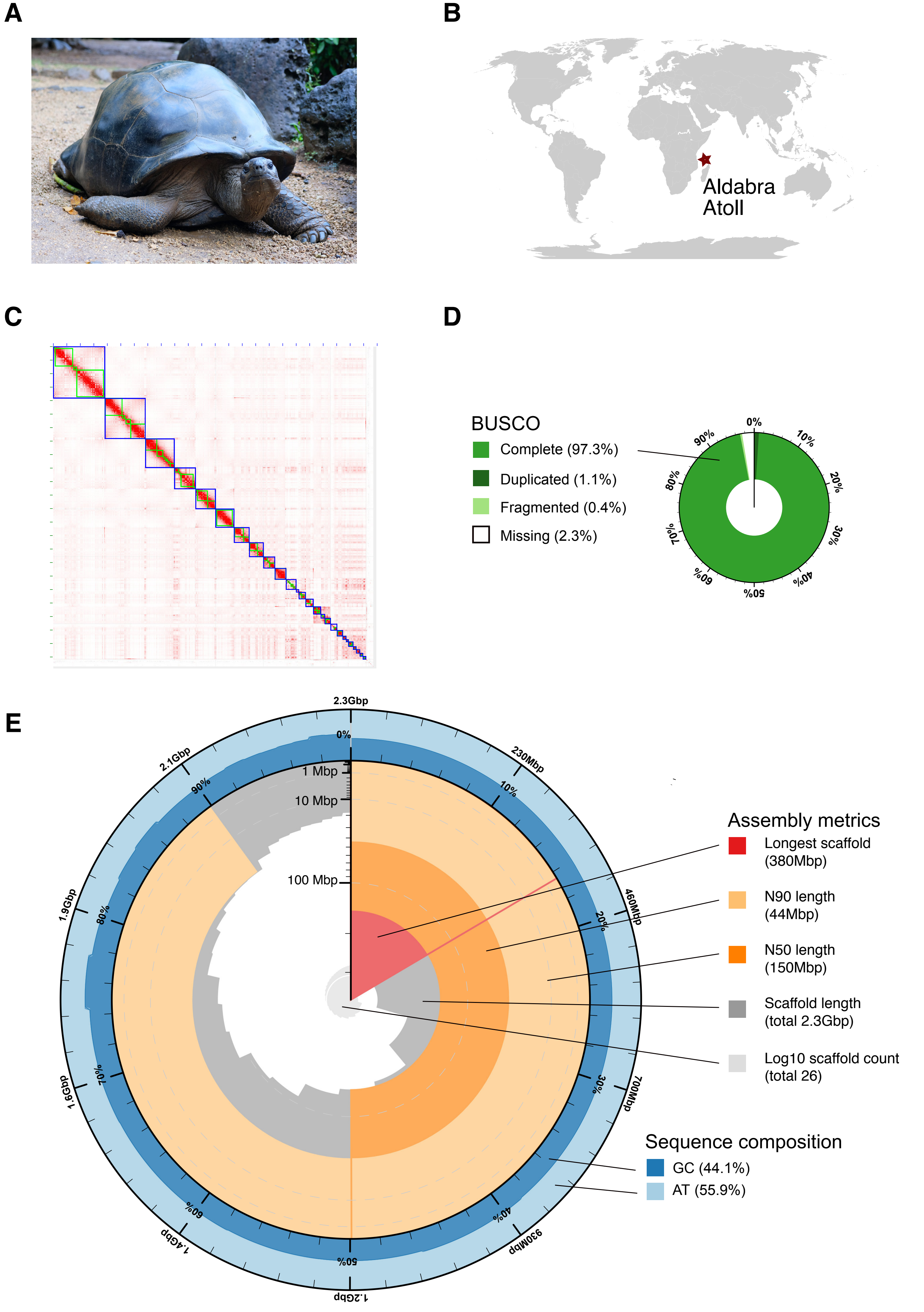

Figure 2

Click here to access/download;Figure;Figure2.pdf

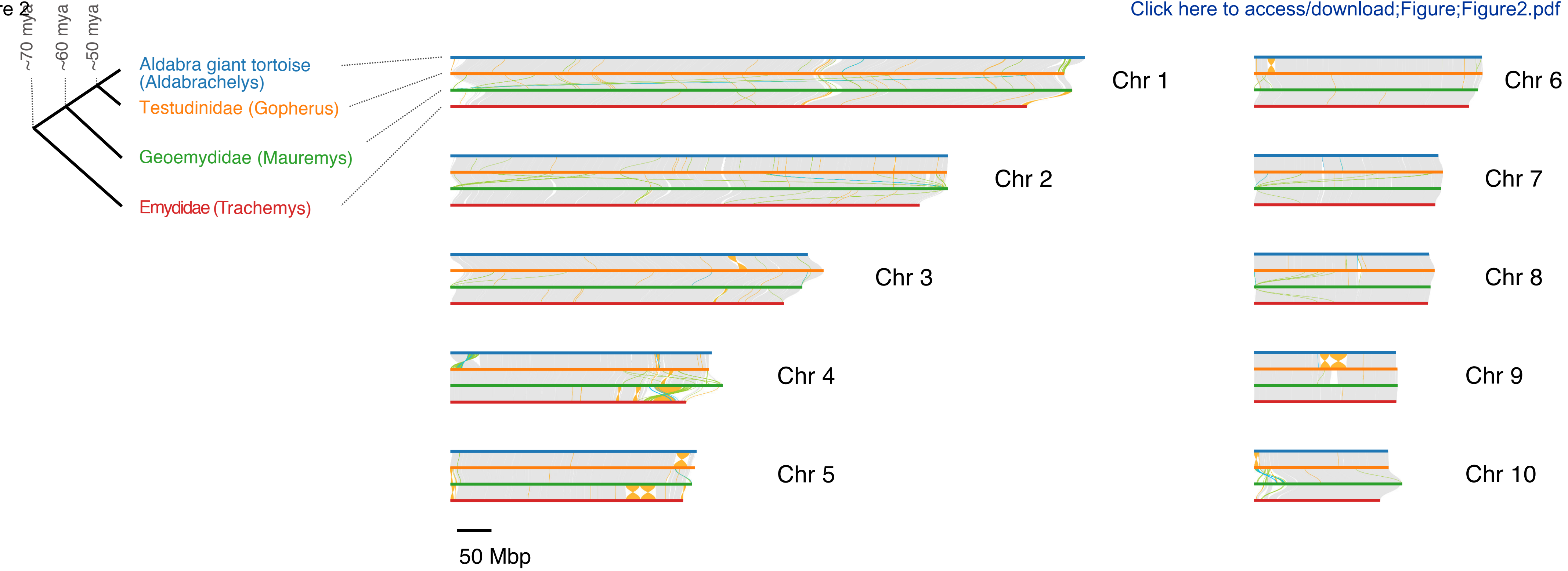

Figure 3 Click here to access/download;Figure;Figure3.pdf

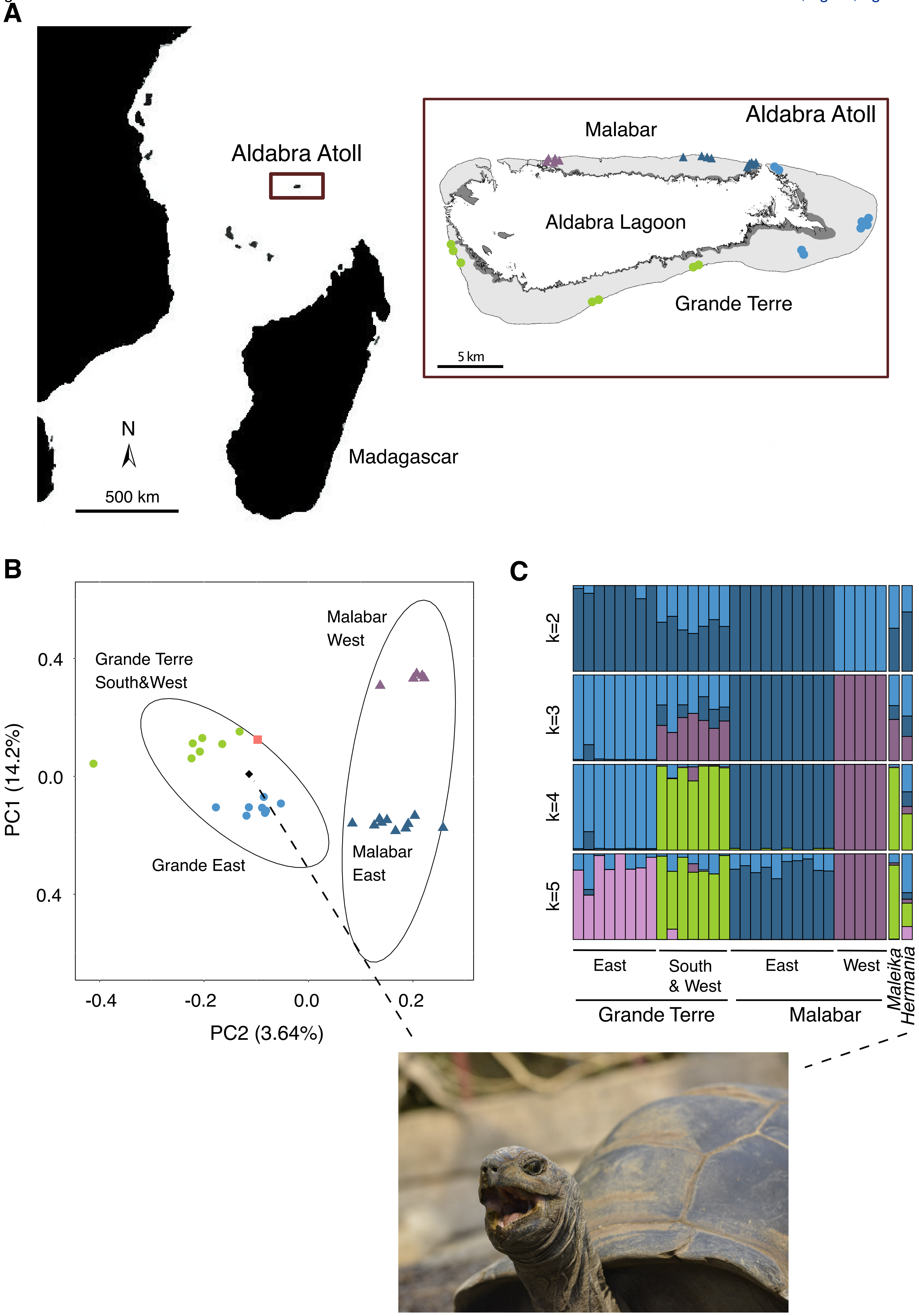

"Hermania" individual - © Leyla Davis, Zoo Zurich

Supplementary Material S1

Click here to access/download
Supplementary Material
Supplementary Material S1.png

Click here to access/download
Supplementary Material
Supplementary Material S2.docx

Click here to access/download
Supplementary Material
Supplementary Material S3.docx

Click here to access/download
Supplementary Material
Supplementary Material S4.xlsx

Click here to access/download
Supplementary Material
Supplementary Material S5.docx

Click here to access/download
Supplementary Material
Supplementary Material S6.docx

Supplementary Material S7

Click here to access/download
Supplementary Material
Supplementary Material S7.png

Click here to access/download
Supplementary Material
Supplementary Material S8.xlsx

Click here to access/download
Supplementary Material
Supplementary Material S9.docx

Supplementary Material S10

Click here to access/download
Supplementary Material
Supplementary Material S10.png
