# Supplementary material for: Chromosome-level genome assembly for the Aldabra giant tortoise enables insights into the genetic health of a threatened population
Source: Gigascience. 2022 Oct 12;11:giac090. doi: 10.1093/gigascience/giac090 (PMC9553416; doi:10.1093/gigascience/giac090)
Supplement: giac090_Supplemental_Files [file giac090_supplemental_files.zip › Supplementary Material S10.docx]

| **Effect Type** | No MAF filter (7,131,506 SNPs) | MAF ≥0.05  (6,651,907 SNPs) |
| --- | --- | --- |
| **High** | 1077 | 630 |
| **Moderate** | 26442 | 20675 |
| **Low** | 46746 | 41424 |
| **Modifier** | 7131506 | 6196752 |
|  |  |  |
