# Supplementary material for: Chromosome-level genome assembly for the Aldabra giant tortoise enables insights into the genetic health of a threatened population
Source: Gigascience. 2022 Oct 12;11:giac090. doi: 10.1093/gigascience/giac090 (PMC9553416; doi:10.1093/gigascience/giac090)
Supplement: giac090_Supplemental_Files [file giac090_supplemental_files.zip › Supplementary Material S2.docx]

| **Genome Statistics** | **HiCanu** | **IPA** | **Hifiasm**  **(default)** | **Hifiasm**  **(with -l 0 option)** |
| --- | --- | --- | --- | --- |
| Contig n | 867 | 2446 | 422 | 703 |
| Contig N50 | 12.6Mbp | 1.6Mbp | 61.5Mbp | 41.4Mbp |
| N Count | 0 | 897 | 0 | 0 |
| Largest contig | 49.8Mbp | 7.8Mbp | 210.3Mbp | 140.8Mbp |
| Total length | 2.3Gbp | 2.3Gbp | 2.4Gbp | 2.5Gbp |
| GC (%) | 44.2 | 44.0 | 44.4 | 44.5 |
