# Supplementary material for: Chromosome-level genome assembly for the Aldabra giant tortoise enables insights into the genetic health of a threatened population
Source: Gigascience. 2022 Oct 12;11:giac090. doi: 10.1093/gigascience/giac090 (PMC9553416; doi:10.1093/gigascience/giac090)
Supplement: giac090_Supplemental_Files [file giac090_supplemental_files.zip › Supplementary Material S3.docx]

| **Type** | **Length (bp)** | **% in genome** | **# of elements** |
| --- | --- | --- | --- |
| Retroelements | 482,092,777 | 20.31 | 1,177,209 |
| LINEs | 293,395,900 | 12.36 | 695,701 |
| LTRs | 137,235,010 | 5.78 | 154,762 |
| SINEs | 51,461,867 | 2.17 | 326,746 |
| DNA elements | 198,183,931 | 8.35 | 642,321 |
| Unclassified | 407,271,311 | 17.16 | 1,902,917 |
| Total interspersed repeats | 1,087,548,019 | 45.82 |  |
