# Supplementary material for: Chromosome-level genome assembly for the Aldabra giant tortoise enables insights into the genetic health of a threatened population
Source: Gigascience. 2022 Oct 12;11:giac090. doi: 10.1093/gigascience/giac090 (PMC9553416; doi:10.1093/gigascience/giac090)
Supplement: giac090_Supplemental_Files [file giac090_supplemental_files.zip › Supplementary Material S5.docx]

| **Species**  **(GenBank Accession No)** |  | | | | |
| --- | --- | --- | --- | --- | --- |
|  |  | **Vertebrate***  **(n = 3354)** | | **Sauropsida***  **(n = 7480)** | |
|  |  | **%** | **#** | **%** | **#** |
| *Aldabrachelys gigantea* | **C** | 93.7 | 3144 | 91.9 | 6874 |
|  | **S** | 90.8 | 3046 | 88.4 | 6613 |
|  | **D** | 2.9 | 98 | 3.5 | 261 |
|  | **F** | 3.1 | 105 | 2.3 | 172 |
|  | **M** | 3.2 | 105 | 5.8 | 434 |
| *Chelonoidis abingdonii*  (GCF_003597395.1) | **C** | 96.9 | 3250 | 97.7 | 7308 |
|  | **S** | 46.1 | 1545 | 46.4 | 3470 |
|  | **D** | 50.8 | 1705 | 51.3 | 3838 |
|  | **F** | 2.5 | 83 | 1.1 | 79 |
|  | **M** | 0.6 | 21 | 1.2 | 93 |
| *Gopherus evgoodei*  (GCF_007399415.2) | **C** | 99.7 | 3342 | 99.3 | 7427 |
|  | **S** | 43.1 | 1445 | 43.8 | 3278 |
|  | **D** | 56.6 | 1897 | 55.5 | 4149 |
|  | **F** | 0.1 | 4 | 0.1 | 10 |
|  | **M** | 0.2 | 8 | 0.6 | 43 |
