# Supplementary material for: Chromosome-level genome assembly for the Aldabra giant tortoise enables insights into the genetic health of a threatened population
Source: Gigascience. 2022 Oct 12;11:giac090. doi: 10.1093/gigascience/giac090 (PMC9553416; doi:10.1093/gigascience/giac090)
Supplement: giac090_Supplemental_Files [file giac090_supplemental_files.zip › Supplementary Material S6.docx]

| **Database** | **#** | **%** |
| --- | --- | --- |
| CDD | 9,210 | 38 |
| Coils | 5,005 | 21 |
| GO | 20,310 | 85 |
| Gene3D | 16,390 | 35 |
| Hamap | 403 | 1 |
| InterPro | 20,310 | 44 |
| MetaCyc | 14,180 | 30 |
| MobiDBLite | 10,961 | 46 |
| PANTHER | 20,383 | 85 |
| PIRSF | 1,449 | 6 |
| PRINTS | 5,458 | 23 |
| Pfam | 18,843 | 79 |
| Phobius | 8,311 | 35 |
| ProSitePatterns | 6,252 | 26 |
| ProSiteProfiles | 11,342 | 47 |
| Reactome | 18,266 | 76 |
| SFLD | 108 | 0 |
| SMART | 10,197 | 43 |
| SUPERFAMILY | 15,477 | 65 |
| SignalPEUK | 2,826 | 12 |
| SignalPGRAMNEGATIVE | 822 | 3 |
| SignalPGRAMPOSITIVE | 1,141 | 5 |
| TIGRFAM | 1,153 | 5 |
| **Genes that have at least one hit from the databases** | 22,554 | 94.1 |
