# Supplementary figures and images for: Chromosome-level genome assembly for the Aldabra giant tortoise enables insights into the genetic health of a threatened population
Source: Gigascience. 2022 Oct 12;11:giac090. doi: 10.1093/gigascience/giac090 (PMC9553416; doi:10.1093/gigascience/giac090)

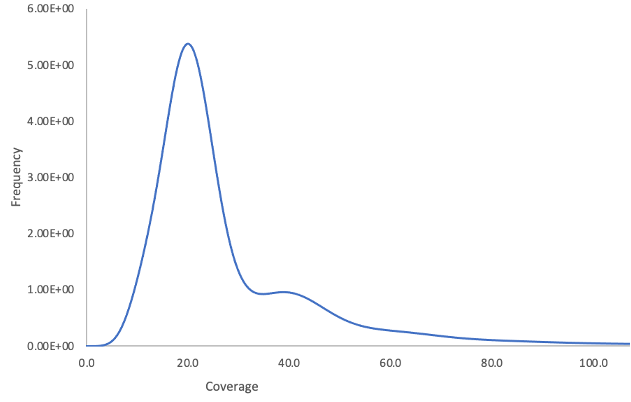

Supplement: giac090_Supplemental_Files [file giac090_supplemental_files.zip › Supplementary Material S1.png]

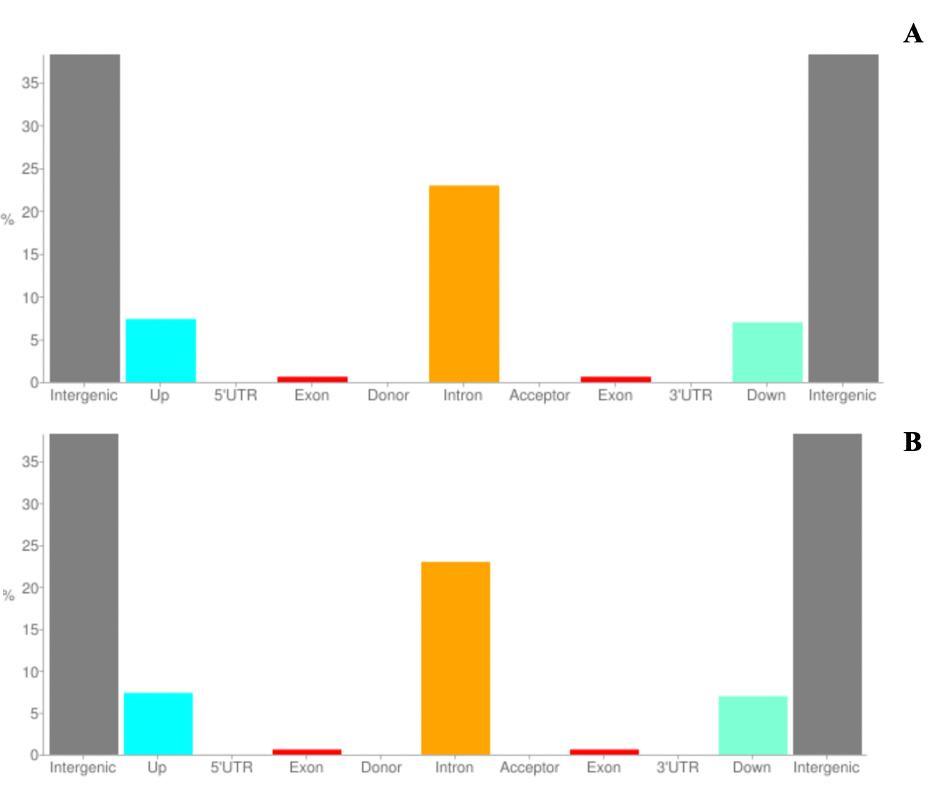

Supplement: giac090_Supplemental_Files [file giac090_supplemental_files.zip › Supplementary Material S11.png]

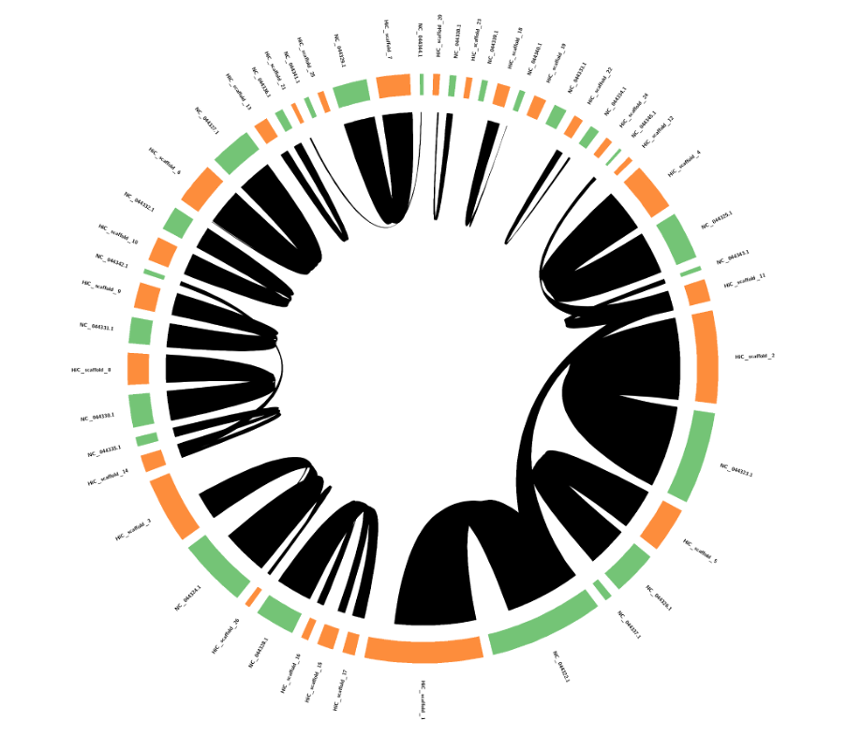

Supplement: giac090_Supplemental_Files [file giac090_supplemental_files.zip › Supplementary Material S7.png]

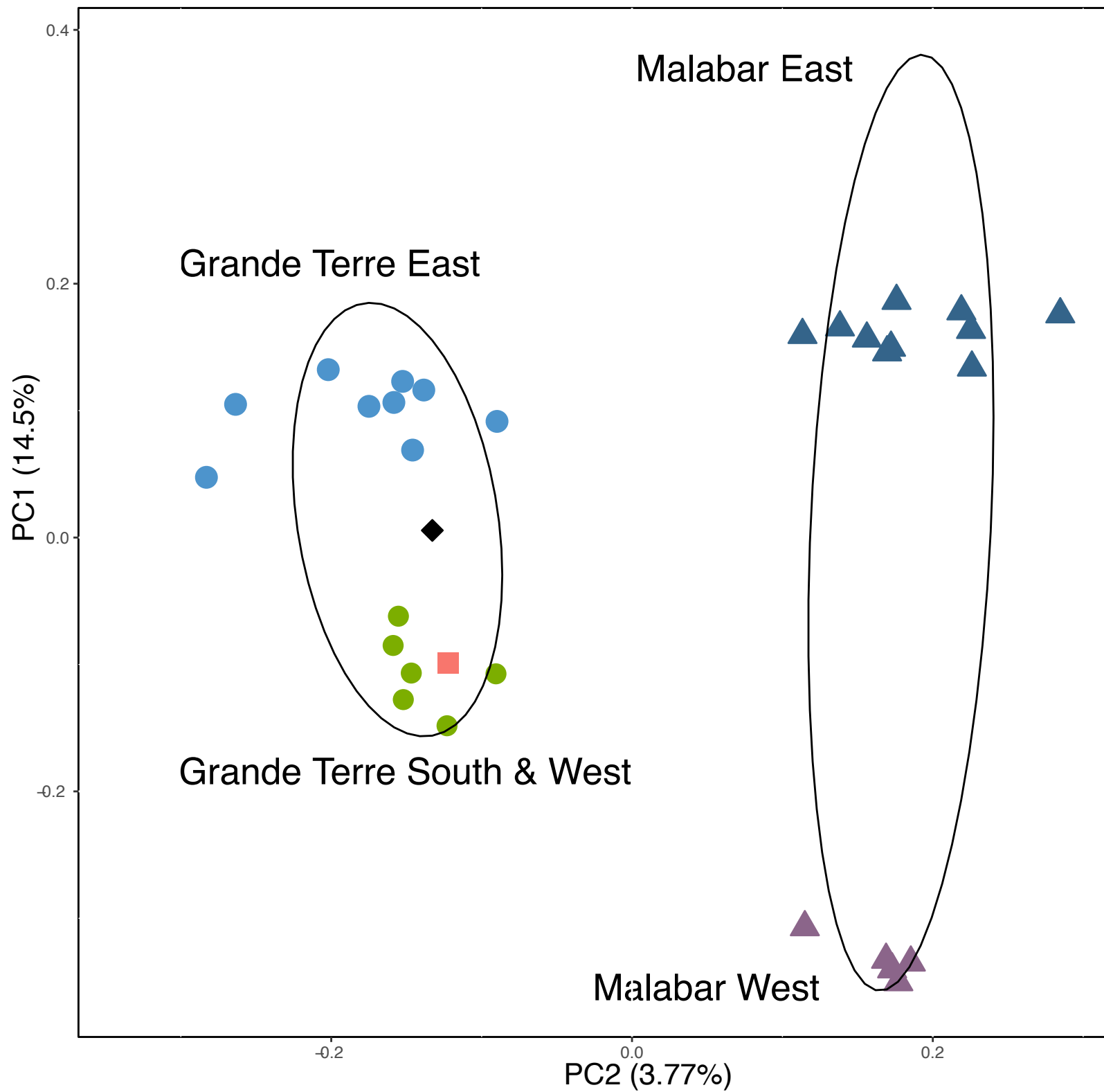

Supplement: giac090_Supplemental_Files [file giac090_supplemental_files.zip › Supplementary Material S9.pdf]
