# Supplementary material for: Chromosome-level genome assembly for the Aldabra giant tortoise enables insights into the genetic health of a threatened population
Source: Gigascience. 2022 Oct 12;11:giac090. doi: 10.1093/gigascience/giac090 (PMC9553416; doi:10.1093/gigascience/giac090)
Supplement: giac090_Response_to_Reviewer_Comments_Original_Submission [file giac090_response_to_reviewer_comments_original_submission.pdf]

Author's Response To Reviewer Comments

Close

Dear Editor,

Thank you very much for the opportunity to revise our manuscript.

We are grateful for the reviewers' helpful feedback and have prepared a point-by-point response for all their comments (including Reviewer 1). The changes in the main text corresponding to the suggestions of Reviewers 1, 2, and 3 are highlighted in green, yellow, and blue, respectively.

Our main modifications are the following:
We performed an additional PCA with a mapping quality of 30, as Reviewer 1 suggested. The new PCA confirmed the results of what we have already reported (a PCA with mapping quality 20) showing the robustness of our results. We, therefore, included the new PCA in the supplementary materials to strengthen our message. To improve the reproducibility of our study, we reported all key parameters for the codes used in this study. Finally, we made minor changes in wording in the main text to improve readability and indicated these changes by coloring the text dark blue. We did not report any new software applications. Therefore, we do not need to receive any new RRID and to include biotoolsID in the manuscript. Neither the data nor the code has been modified, so we did not update the public versions.

We hope that you will find the new version of this manuscript suitable for publication.

On behalf of all the authors,
Dr. F. Gözde Cilingir & Dr. Christine Grossen

Reviewer reports:

Reviewer #2: This manuscript by Çilingir et al. describes the sequencing and assembly at chromosomal level of a reference genome for the Aldabra tortoise. Using this reference, the work appraises the genomic diversity of Aldabra turtles by re-sequencing. The authors use these data to provide information on the genomic diversity of Aldabra tortoises. They also provide an ab initio annotation of the assembly. I find the manuscript interesting and useful to inform conservation efforts. The manuscript is clear and well written, with detailed reporting on sample collection and treatment, as well as on bioinformatic methods. Raw and processed sequencing data are available in standard formats. I would only suggest a few minor edits:

+Response: We thank Reviewer 2 for their helpful comments. The changes in the main text regarding their comments can be found highlighted in yellow.

- Since the manuscript mentions the importance of genomic diversity to aid conservation efforts, it would be useful to compare the genomic diversity of Aldabra tortoises to that of other endangered species.

+Response: We now mention diversity estimates from a few other species with a history of a severe bottleneck, such as the Amur tiger, Eastern Mountain Gorilla, and Giant Panda, as well as two endangered turtle species Pinta Island tortoise and Reeve's Turtle (lines 151-157).

-In Figure 3, the picture of the Hermania individual connects two different panels. While I understand the information conveyed in this picture, I believe that panels should be independent. If the authors

wish to keep this flow of information, I would advise to merge B and C.

+Response: We prefer to keep B and C as separate panels since they represent two different analyses. Instead, we have created a panel D for the photograph and edited the legend accordingly.

-Also in Figure 3, it seems unclear whether the colors of panel C are related to those of panels A and B. My understanding is that they are not, but the correlation in the case k=4 seems too good to be random. The authors should clarify whether this is on purpose and how it was achieved.

+Response: We did indeed use the same colors for the different groups where possible, and the colors for k=4 correspond to the colors in panel A and panel B. This is now specified in the figure legend.

-Supplementary Figure 10 is referenced in the text when discussing deleterious variants. The figure does not contain information on those kinds of variants. The authors may consider adding this information, as the second panel is similar to the first and does not add much information.

+Response: We agree that supplementary material 10 had additional information to the deleterious variants that we reported; therefore, we included "see also" where we refer to the supplementary material information.

Reviewer #3: The manuscript describes the sequencing, assembly, annotation, and utility of an Aldabra tortoise genome. It is mostly well written and the assembly will be a useful addition to complete high quality genomes for tortoises (and turtles and vertebrates in general). There are some confusing sections, and some methods and analyses should be explained in more detail, which would be useful to the genomics community. I have a few comments on the manuscript the authors may take into consideration.

+Response: We thank Reviewer 3 for their valuable comments. The changes in the main text regarding their comments can be found highlighted in light blue.

Abstract Background:

-I assume when "vulnerable" status of Aldabra is also according to IUCN, but the authors should specify.

+Response: Yes, "vulnerable" refers to the IUCN Red List status of the species. The suggested addition has been made.

Main Background:

-A few grammar/sentence structure issues on page 3:
** genomic technologies are already important, not "becoming" increasingly important

+Response: The sentence was changed to "…genomic technologies are an important tool for conservation researchers."

** "The establishment" through "changing environments" is a run-on sentence and difficult to read and understand. The authors should consider reworking this sentence, and the following sentence as well.

+Response: We reworked this sentence by dividing it into two.

** "Thanks to" is pretty informal, the authors should more precisely describe the relationship between the number of genomes and consortia.

+Response: We replaced "thanks to" with "due to."

** "(or tortoises)" should be just ("tortoises")

+Response: This change has been made.

-If there is an IUCN report on these tortoises claiming their conservation status, the authors should cite the paper or the web link.

+Response: There are 14 Galapagos giant tortoise species that are listed in the IUCN Red List v2.3. Because we do not include any species-specific information, we used the general citation of the IUCN Red List as recommended here:
https://www.iucnredlist.org/about/citationinfo

-I assume when "vulnerable" status of Aldabra is also according to IUCN, but the authors should specify.

+Response: That is correct. We added the citation for Aldabra giant tortoise-specific weblink where necessary.

-"Exceptional life history traits" - these are not known to the reader and the authors should cite some specifics here.

+Response: We now mention a few of these exceptional traits and added a citation. The new sentence is "Genomes of giant tortoises may harbor clues to their exceptional life-history traits such as long life span[13] and gigantism[14–16]"

-The authors should describe the geographic location of the Mauritian istels of Ile Aux Aigrettes and Round Island. Where are they?

+Response: These islands are in Mauritius. To clarify, we changed the sentence to "Aldabrachelys gigantea has been introduced to three islands belonging to Mauritius, including Ile aux Aigrettes, Round Island, and Rodrigues[24]."

Nuclear Genome Assembly, Contamination Scan and Evaluation:

-What does DIAMOND do? The authors should explain what it is used for.

+Response: We changed the corresponding sentence to "Each segment was searched against the full NCBI non-redundant protein database by running diamond, a tool that performs protein alignments against reference databases, with the blastx option."

-What is defined as an "external source"? It is not clear what the cutoff values "30, 0.0001, 70%" are referring to. What parameters are these for?

+Response: By using "external source," we meant contamination. Bitscore and evalue are blast parameters, GC content refers to the segment's GC content. To make our message clear, we divided the original sentence and added an explanation of the cutoff values.

"Each segment was searched against the full NCBI non-redundant protein database by running diamond v2.0.9(DIAMOND, RRID:SCR_016071)[46,47], a tool that performs protein alignments against reference databases, with the blastx option. We considered a segment to be a likely contaminant based on the blast bitscore (>30), e-value (>0.0001), and the segment's GC content (>70%). None of the blastx hits passed any of these cutoffs, and hence none of them was considered a significant match and potential contaminant."

-"the most probable sources of contamination human" should be "the most probable sources of contamination from the human"

+Response: This change has been made.

-The authors compared kmer profiles between human and tortoise genomes, but how were they determined to be "distinct"? Is there a test metric or vidual inspection of histograms that were used? The kmer analysis should be explained in more detail.

+Response: We acknowledge that this may not have been clear enough. For k-mer-based contaminant detection, we used the sect tool (SEquence Coverage estimator tool) within the software KAT. Basically, this tool estimates the coverage of each sequence in a file using k-mers from another sequence file. In our case, this tool counted k-mers in a putative contaminant genome (here, human genome and Aldabra giant tortoise mitogenome) and applied them to the draft Aldabra giant tortoise assembly sequences. Contigs that potentially belong to the contaminant are outputted with a score named

“%_non_zero_corrected” indicating the percentage of the sequence which has a k-mer coverage greater than 1. Assuming the contaminant is the exact same species as that found in the queried assembly, very high percentage scores (>90%) would be expected. Moderate scores (20-80%) might indicate either some shared content or chimeric sequences. In our contamination detection analysis, both the human genome and Aldabra giant tortoise mitochondrial genome resulted in scores of <0.01% per each contig within the Aldabra draft genome assembly. Hence, we found no evidence of contamination.

We updated the corresponding parts as:
“The average k-mer frequency of each contig in the draft assembly was compared with the potential contamination source using the tool sect in the software KAT v2.4.1 (KAT, RRID:SCR_016741)[48,49]). Less than 0.01% of all contigs in the draft assembly showed k-mer statistics indicative of potential contamination (a validated k-mer coverage >1) by either source.‟

Repetitive Element Analysis:

- How do the repeat analysis results compare to other chelonians? This information should be available for numerous species, the authors should place the results here in the context of what is typical for a turtle.

+Response: Repeat analysis results are available for many of the published genomes, but the differences in methodologies for repeat identification make it hard to have comparable results. Still, to give some comparison, we have included repeat ratios of Chelonia mydas, Gopherus evgoodei, Chrysemys picta belli and Trachemys scripta elegans, estimated by Simison et al. (2020), where a repeat identification pipeline similar to our pipeline was run. The following sentence was added at the end of the paragraph:

“The repeat content of the A. gigantea genome was found to be slightly higher than the repeat contents of the green sea turtle (Chelonia mydas) (41.67%), Goode's thornscrub tortoise (Gopherus evgoodei) (41.67%), painted turtle (Chrysemys picta belli) (42%), and red-eared slider (Trachemys scripta elegans) (45%) genomes [68].”

Gene Prediction and annotation:

-"Using pre-trained parameters yielded more complete annotations compared to training with extrinsic evidence" - how was this determined? Did the authors look at the cumulative distribution frequencies of Annotation Editing Distances for each annotation? Or compare BUSCOs on each final protein dataset?

+Response: Thank you for pointing this out. We compared BUSCOs on each final protein dataset, and we included this information where necessary. We changed the sentence to “Using pre-trained parameters yielded more complete annotations compared to training with extrinsic evidence (i.e., RNA-seq and protein data) as assessed by BUSCO protein completeness analyses.”

Synteny Analysis:

-"Is in agreement with previous studies" - these studies should be cited.

+Response: We cited two studies in addition to another paper that briefly summarizes these studies.

-"Eventually, the high ratio of synteny revealed by our complementary collinnearity analysis supported our findings on the chromosomal synteny analysis." - I am not sure what this last sentence means.

+Response: The first paragraph in this section is about the chromosomal synteny analysis, and the second paragraph is about the complementary collinearity analysis we performed. In the last sentence, we wanted to emphasize that the results of both analyses are in line with each other. For clarification, we changed the sentence to:

“Both synteny analysis approaches were providing a consistent picture of high collinearity.”

Sample collection for low coverage whole-genome resequencing:

- The first paragraph feels out of place and may be better put in the Background or Discussion.

+Response: We prefer not to move the first paragraph of this subsection as we believe that the whole section is more coherent this way.

Data Filtering, alignment, and genotype likelihood estimation:

- It is not made clear why having a reference genome assembly enhances these analyses. Perhaps an estimation of runs of homozygosity can capitalize on the contiguity of the high quality assembly in order to assess inbreeding for a vulnerable species.

+Response: We realize that it was not yet clear enough how this new reference genome will help future analysis. We added to the conclusion the gained possibility to estimate inbreeding levels via runs of homozygosity. However, with our low-coverage resequencing data, we do not feel comfortable doing analyses of runs of homozygosity. We think the results would not be reliable enough.

- The use of "also" feels out of place; there isn't a previous statement of the benefits of a high-quality Aldabra genome.

+Response: We cannot spot any “also” words within the section Data Filtering, alignment, and genotype likelihood estimation. We checked all subsequently used “also” words and could not detect any connections with the benefits of a high-quality Aldabra genome.

Conclusions:

- Page 20- If the authors are going to mention aging and gigantism here, they should introduce it in the background (rather than alluding to "special life history traits" that make this species worth studying). Gigantism wasn't defined anywhere else in the paper.

+Response: Based on a previous comment by the reviewer, we have added a sentence in the Background section.

The authors should make the NCBI accession numbers public before publication.

+Response: All the raw sequencing files, the assembly, and annotation are already uploaded to public repositories. They will be open to accession upon the acceptance of the manuscript, as stated in the Data Availability section.

Reviewer #1

We thank reviewer 1 for their helpful comments. All newly added parts based on their suggestions were highlighted in green.

Main points:
1. Were import/export permits required? If so, please provide numbers

+Response: We thank the reviewer for pointing out the import/export permits. We received an export permit from Seychelles with permit no A1457. As for the import permit within Switzerland, our department within the University of Zurich has a general import permit for CITES Appendix II listed non-avian reptiles. We updated the main text in regard to import/export permits: “An export permit was issued by the Ministry of Agriculture, Climate Change and Environment, Republic of Seychelles (permit #A1457), and an import permit was granted by the Federal Food Safety and Veterinary Office of Switzerland to the Department of Evolutionary Biology and Environmental Studies, University of Zurich (permit #19DB000064/22-AS).”

2. I don't see some of the summary data files provided (e.g., filtered VCF). I think these should be provided for this resource to be readily usable.

+Response: We previously uploaded two filtered VCF files to the database of GigaScience, one with variants with MAF ≥ 0.01 and another with MAF ≥ 0.05 (file names: variant_annotation_maf01.vcf.gz, variant_annotation_maf05.vcf.gz)

3. Many of the methods (especially the first half) do not include relevant flag/parameter information. All default parameters need to be clearly stated or provided as script files in the supplementary. Otherwise none of this is reproducible.

+Response: We have added all key parameters where they were missing, especially in the first half.

Minor points:
P3- "the establishment…changing environments" -this sentence is too long and conveying too many ideas

+Response: Reviewer 2 pointed out the same problem. We have divided this long sentence into two parts.

P12 - "The resulting gene model…" - the gene count is expected, but 39kbp is extremely long for a gene model relative to other chelonians if I'm not mistaken. Please explain this result

+Response: We are not sure if it was maybe unclear that the gene length includes introns. We do not think that 39 Kbp is very long. For instance, the mean gene length is larger in Gopherus evgoodei (48 Kbp) Chelonoidis niger abingdonii (45 Kbp), and Chelonia mydas (47 Kbp). We have clarified that this includes introns and added the above numbers for comparison:

"The resulting gene model set consisted of 23,953 protein-coding genes with a mean gene length of 39,458 bp (including introns) and an average of nine exons per coding sequence (Table 1). The mean gene length is smaller compared to genes of other turtles such as G. evgoodei (48 Kbp; NCBI RefSeq: GCF_007399415.2) C. n. abingdonii (45 Kbp; NCBI RefSeq: GCF_003597395.1) and C. mydas (47 Kbp; NCBI RefSeq: GCF_015237465.2)."

P17 - assuming this is a PHRED score, isn't a mapping quality score of 20 quite low (1% error)? Please justify or compare against a more restrictive (e.g., >30) dataset

+Response: A mapping quality of 20 conforms with the general recommendations for analyses with ANGSD (Lou et al., 2021). But we also ran a PCA with a minimum mapping quality of 30, which we now show in Supplementary Material S9. This new analysis confirms the result shown in Figure 3.

Literature Cited
Lou, R. N., Jacobs, A., Wilder, A. P., & Therkildsen, N. O. (2021). A beginner's guide to low-coverage whole genome sequencing for population genomics. Molecular Ecology, 30, 5966– 5993. https://doi.org/10.1111/mec.16077.

Table 1 - I suggest adding average gene length since it's reported in the text

+Response: Gene length has been added to Table 1.

Table 2 - Gopherus agassizii had a chromosome-level assembly published in 2020 that is missing from this table. If the table is limited to those on NCBI then this should be stated.

+Response: To the best of our knowledge, the Gopheurs agassizzii reference genome is scaffold-level, not chromosome-level, assembled with a contig N50 of 44 Kbp and a scaffold N50 of 228 Kbp. Because the genome is not chromosome-level assembled, we did not include its details in Table 2. Assembly details in ENA can be found here:
https://www.ebi.ac.uk/ena/browser/view/GCA_002896415.1

Figure 1 - I wonder if this is the best use of space, the figure is quite large and conveys relatively little information. While attractive, I also wonder if the sequence composition in panel E is worth showing since it's consistent (and doesn't really matter in the first place). Can the authors change the external ring to something more useful, like gene density/distribution relative to TAD domains?

+Response: The Circos plot in Figure 1 is a description of the draft assembly statistics, not the annotation performance. We think it is more consistent to leave it as is.

Figure 2 - genus names should be italicized

+Response: This change has been made.

SM S7 - please upload a pdf instead so the figure labels are readable (the png is pixelated)

+Response: Pdf versions of all the figures were uploaded.

Finally, if this goes back out to review, *PLEASE* use line numbers on your submission, it is very cumbersome to review without them.

+Response: It was stated on the website of GigaScience that line numbers will be automatically added to the .docx files uploaded to their system. Something must have gone wrong. Our apologies. We added line numbers to the revised manuscript.

Close
