# Supplementary material for: Chromosome-level genome assembly for the Aldabra giant tortoise enables insights into the genetic health of a threatened population
Source: Gigascience. 2022 Oct 12;11:giac090. doi: 10.1093/gigascience/giac090 (PMC9553416; doi:10.1093/gigascience/giac090)
Supplement: giac090_Reviewer_2_Report_Original_Submission — VÃctor Quesada -- 6/6/2022 Reviewed [file giac090_reviewer_2_report_original_submission.pdf]

Reviewer Report

Title: Chromosome-level genome assembly for the Aldabra giant tortoise enables insights into the genetic health of a threatened population

Version: Original Submission **Date:** 6/6/2022

Reviewer name: VÃctor Quesada

Reviewer Comments to Author:

This manuscript by Ã‡ilingir et al. describes the sequencing and assembly at chromosomal level of a reference genome for the Aldabra tortoise. Using this reference, the work appraises the genomic diversity of Aldabra turtles by re-sequencing. The authors use these data to provide information on the genomic diversity of Aldabra tortoises. They also provide an ab initio annotation of the assembly.
I find the manuscript interesting and useful to inform conservation efforts. The manuscript is clear and well written, with detailed reporting on sample collection and treatment, as well as on bioinformatic methods. Raw and processed sequencing data are available in standard formats. I would only suggest a few minor edits:
-Since the manuscript mentions the importance of genomic diversity to aid conservation efforts, it would be useful to compare the genomic diversity of Aldabra tortoises to that of other endangered species.
-In Figure 3, the picture of the Hermania individual connects two different panels. While I understand the information conveyed in this picture, I believe that panels should be independent. If the authors wish to keep this flow of information, I would advise to merge B and C.
-Also in Figure 3, it seems unclear whether the colors of panel C are related to those of panels A and B. My understanding is that they are not, but the correlation in the case k=4 seems too good to be random. The authors should clarify whether this is on purpose and how it was achieved.
-Supplementary Figure 10 is referenced in the text when discussing deleterious variants. The figure does not contain information on those kinds of variants. The authors may consider adding this information, as the second panel is similar to the first and does not add much information.

Level of Interest

Please indicate how interesting you found the manuscript: Choose an item.

Quality of Written English

Please indicate the quality of language in the manuscript: Choose an item.

Declaration of Competing Interests

Please complete a declaration of competing interests, considering the following questions:

- Have you in the past five years received reimbursements, fees, funding, or salary from an organisation that may in any way gain or lose financially from the publication of this manuscript, either now or in the future?
- Do you hold any stocks or shares in an organisation that may in any way gain or lose financially from the publication of this manuscript, either now or in the future?
- Do you hold or are you currently applying for any patents relating to the content of the manuscript?
- Have you received reimbursements, fees, funding, or salary from an organization that holds or has applied for patents relating to the content of the manuscript?
- Do you have any other financial competing interests?
- Do you have any non-financial competing interests in relation to this paper?

If you can answer no to all of the above, write 'I declare that I have no competing interests' below. If your reply is yes to any, please give details below.

I declare that I have no competing interests

I agree to the open peer review policy of the journal. I understand that my name will be included on my report to the authors and, if the manuscript is accepted for publication, my named report including any attachments I upload will be posted on the website along with the authors' responses. I agree for my report to be made available under an Open Access Creative Commons CC-BY license (http://creativecommons.org/licenses/by/4.0/). I understand that any comments which I do not wish to be included in my named report can be included as confidential comments to the editors, which will not be published.

Choose an item.

To further support our reviewers, we have joined with Publons, where you can gain additional credit to further highlight your hard work (see: https://publons.com/journal/530/gigascience). On publication of this paper, your review will be automatically added to Publons, you can then choose whether or not to claim your Publons credit. I understand this statement.

Yes Choose an item.
