# Supplementary material for: Chromosome-level genome assembly for the Aldabra giant tortoise enables insights into the genetic health of a threatened population
Source: Gigascience. 2022 Oct 12;11:giac090. doi: 10.1093/gigascience/giac090 (PMC9553416; doi:10.1093/gigascience/giac090)
Supplement: giac090_Reviewer_3_Report_Original_Submission — Marc Tollis -- 6/13/2022 Reviewed [file giac090_reviewer_3_report_original_submission.pdf]

Reviewer Report

Title: Chromosome-level genome assembly for the Aldabra giant tortoise enables insights into the genetic health of a threatened population

Version: Original Submission **Date:** 6/13/2022

Reviewer name: Marc Tollis

Reviewer Comments to Author:

The manuscript describes the sequencing, assembly, annotation, and utility of an Aldabra tortoise genome. It is mostly well written and the assembly will be a useful addition to complete high quality genomes for tortoises (and turtles and vertebrates in general). There are some confusing sections, and some methods and analyses should be explained in more detail, which would be useful to the genomics community. I have a few comments on the manuscript the authors may take into consideration.
Abstract Background:
-I assume when "vulnerable" status of Aldabra is also according to IUCN, but the authors should specify.
Main Background:
-A few grammar/sentence structure issues on page 3:
** genomic technologies are already important, not "becoming" increasingly important
** "The establishment" through "changing environments" is a run-on sentence and difficult to read and understand. The authors should consider reworking this sentence, and the following sentence as well.
** "Thanks to" is pretty informal, the authors should more precisely describe the relationship between the number of genomes and consortia.
** "(or tortoises)" should be just ("tortoises")
-If there is an IUCN report on these tortoises claiming their conservation status, the authors should cite the paper or the web link.
-I assume when "vulnerable" status of Aldabra is also according to IUCN, but the authors should specify.
-"Exceptional life history traits" - these are not known to the reader and the authors should cite some specifics here.
-The authors should describe the geographic location of the Mauritian istels of Ile Aux Aigrettes and Round Island. Where are they?
Nuclear Genome Assembly, Contamination Scan and Evaluation:
-What does DIAMOND do? The authors should explain what it is used for.
-What is defined as an "external source"? It is not clear what the cutoff values "30, 0.0001, 70%" are referring to. What parameters are these for?
-"the most probable sources of contamination human" should be "the most probable sources of contamination from the human"
-The authors compared kmer profiles between human and tortoise genomes, but how were they determined to be "distinct"? Is there a test metric or vidual inspection of histograms that were used? The kmer analysis should be explained in more detail.
Repetitive Element Analysis:

- How do the repeat analysis results compare to other chelonians? This information should be available for numerous species, the authors should place the results here in the context of what is typical for a turtle.
Gene Prediction and annotation:
-"Using pre-trained parameters yielded more complete annotations compared to training with extrinsic evidence" - how was this determined? Did the authors look at the cumulative distribution frequencies of Annotation Editing Distances for each annotation? Or compare BUSCOs on each final protein dataset?
Synteny Analysis:
-"Is in agreement with previous studies" - these studies should be cited.
-"Eventually, the high ratio of synteny revealed by our complementary collinnearity analysis supported our findings on the chromosomal synteny analysis." - I am not sure what this last sentence means.
Sample collection for low coverage whole-genome resequencing:
- The first paragraph feels out of place and may be better put in the Background or Discussion.
Data Filtering, alignment, and genotype likelihood estimation:
- It is not made clear why having a reference genome assembly enhances these analyses. Perhaps an estimation of runs of homozygosity can capitalize on the contiguity of the high quality assembly in order to assess inbreeding for a vulnerable species.
3. The use of "also" feels out of place; there isn't a previous statement of the benefits of a high-quality Aldabra genome.
Conclusions:
- Page 20- If the authors are going to mention aging and gigantism here, they should introduce it in the background (rather than alluding to "special life history traits" that make this species worth studying). Gigantism wasn't defined anywhere else in the paper.
The authors should make the NCBI accession numbers public before publication.

Level of Interest

Please indicate how interesting you found the manuscript: Choose an item.

Quality of Written English

Please indicate the quality of language in the manuscript: Choose an item.

Declaration of Competing Interests

Please complete a declaration of competing interests, considering the following questions:

- Have you in the past five years received reimbursements, fees, funding, or salary from an organisation that may in any way gain or lose financially from the publication of this manuscript, either now or in the future?

- Do you hold any stocks or shares in an organisation that may in any way gain or lose financially from the publication of this manuscript, either now or in the future?
- Do you hold or are you currently applying for any patents relating to the content of the manuscript?
- Have you received reimbursements, fees, funding, or salary from an organization that holds or has applied for patents relating to the content of the manuscript?
- Do you have any other financial competing interests?
- Do you have any non-financial competing interests in relation to this paper?

If you can answer no to all of the above, write 'I declare that I have no competing interests' below. If your reply is yes to any, please give details below.

I declare that I have no competing interests.

I agree to the open peer review policy of the journal. I understand that my name will be included on my report to the authors and, if the manuscript is accepted for publication, my named report including any attachments I upload will be posted on the website along with the authors' responses. I agree for my report to be made available under an Open Access Creative Commons CC-BY license (http://creativecommons.org/licenses/by/4.0/). I understand that any comments which I do not wish to be included in my named report can be included as confidential comments to the editors, which will not be published.

Choose an item.

To further support our reviewers, we have joined with Publons, where you can gain additional credit to further highlight your hard work (see: https://publons.com/journal/530/gigascience). On publication of this paper, your review will be automatically added to Publons, you can then choose whether or not to claim your Publons credit. I understand this statement.

Yes Choose an item.
